# Towards safer anti-inflammatory therapy: synthesis of new thymol–pyrazole hybrids as dual COX-2/5-LOX inhibitors

**DOI:** 10.1080/14756366.2022.2147164

**Published:** 2022-11-21

**Authors:** Mostafa M. M. El-Miligy, Ahmed K. Al-Kubeisi, Mohamed G. Bekhit, Saad R. El-Zemity, Rasha A. Nassra, Aly A. Hazzaa

**Affiliations:** a Pharmaceutical Chemistry Department, Faculty of Pharmacy, Alexandria University, Alexandria, Egypt; b Pharmacy Department, Alnukhba University College, Baghdad, Iraq; c Pharmaceutical Chemistry Department, Faculty of Pharmacy, Pharos University in Alexandria, Alexandria, Egypt; d Department of Chemistry and Technology of Pesticides, Faculty of Agriculture, Alexandria University, Alexandria, Egypt; e Medical Biochemistry Department, Faculty of Medicine, Alexandria University, Alexandria, Egypt

**Keywords:** Thymol, pyrazole, anti-inflammatory, COX-2, 5-LOX

## Abstract

New thymol − 1,5-disubstitutedpyrazole hybrids were synthesised as dual COX-2/5-LOX inhibitors. Compounds **8b**, **8g**, **8c,** and **4a** displayed *in vitro* inhibitory activity against COX-2 (IC_50 _= 0.043, 0.045, 0.063, and 0.068 µM**)** nearly equal to celecoxib (IC_50 _= 0.045 µM**)** with high SI (316, 268, 204, and 151, respectively) comparable to celecoxib (327). All target compounds, **4a–c** and **8a–i**, showed *in vitro* 5-LOX inhibitory activity higher than reference quercetin. Besides, they possessed *in vivo* inhibition of formalin-induced paw oedema higher than celecoxib. In addition, compounds **4a, 4b, 8b,** and **8g** showed superior gastrointestinal safety profile (no ulceration) as celecoxib and diclofenac sodium in the population of fasted rats. In conclusion, compounds **4a**, **8b,** and **8g** achieved the target goal. They elicited *in vitro* dual inhibition of COX-2/5-LOX higher than celecoxib and quercetin, *in vivo* potent anti-inflammatory activity higher than celecoxib and *in vivo* superior gastrointestinal safety profile (no ulceration) as celecoxib.

## Graphical Abstract

**Figure:**
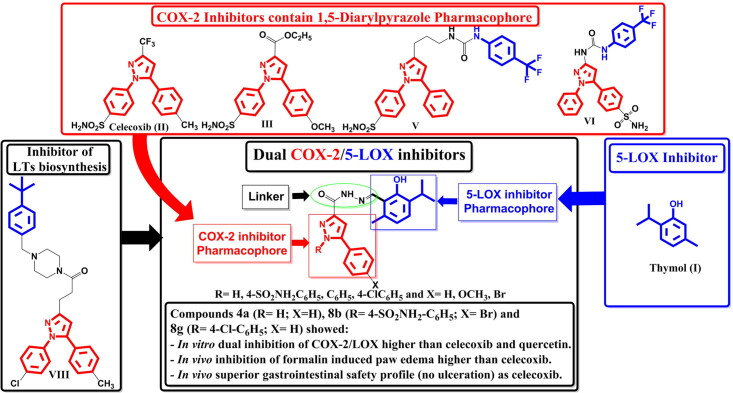

## Introduction

**Figure 1.**
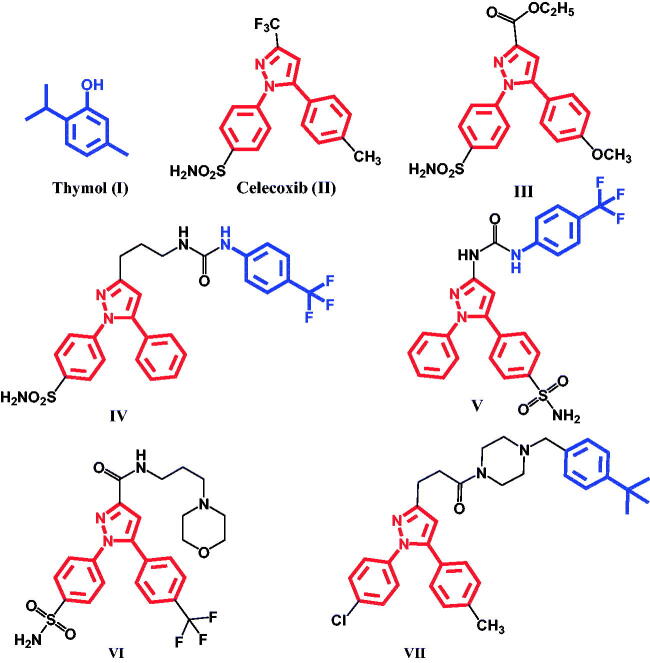
Structures of compounds having COX-2 or 5-LOX inhibitory activities.

Inflammation includes variety of mechanisms and release of mediators which lead to drawbacks in the cardiovascular system and the renal apparatus^1^. Non-steroidal anti-inflammatory drugs (NSAIDs) are widely used for the treatment of inflammation, pain, fever, and arthritis. NSAIDs decrease prostaglandins production *via* inhibition of cyclooxygenase (COX) enzymes. Classical NSAIDs inhibit both COX-1 and COX-2 enzymes which lead to anti-inflammatory activity as well as their side effects^2–6^. Accordingly, several selective COX-2 inhibitors such as celecoxib, valdecoxib, and rofecoxib have been developed and were approved for clinical use due to their low gastrointestinal side effects. However, their long-term use has been reported to cause cardiovascular side effects and was withdrawn from the market^7–10^. The inhibition of COX-1/COX-2 result in an increased formation of leukotrienes (LTs) through the lipoxygenase (LOX) pathway^11^. Overproduction of LTs induces asthmatic problems, gastric damage, and ulceration^12–17^. Moreover, 5-LOX has been related to several undesirable physiological effects which are involved in the progression of inflammation, osteoarthritis, and asthma^18–20^. Accordingly, dual inhibition of COX-2/5-LOX could provide anti-inflammatory effects with reduced side effects ^21^. Furthermore, the natural phenol derivative, thymol **I**, was proved to have COX-2 inhibition and anti-inflammatory activities^22–24^. Moreover, thymol was found to abolish the activity of 5-LOX in human monocytic (THP-1) cell line^25^. On the other hand, several 1,5-diaryl pyrazoles were reported to have remarkable anti-inflammatory activity comparable to the lead celecoxib **II.** The anti-inflammatory activity of celecoxib remained in the 3-carboxylate derivative **III**^26^. Moreover, linking phenyl urea to pyrazole in compounds **IV** and **V** resulted in dual inhibition of COX-2 and Soluble Epoxide Hydrolase with favourable cardiovascular profile than celecoxib^27^^,^^28^. Furthermore, hybridisation of 1,5-diarylpyrazole with morpholine produced dual COX-2/5-LOX inhibitor with low toxicity, compound **VI**^29^. In addition, replacement of morpholine with its bioisostere piperazine and linking it to 4-tert-butylbenzyl moiety in compound **VII** showed potent inhibition of LT biosynthesis in activated human neutrophils^30^ (Figure 1). Moreover, various N-acylhydrazone derivatives were reported to have analgesic, anti-inflammatory, and COX-2 inhibitory activities^31–34^.

**Figure 2.**
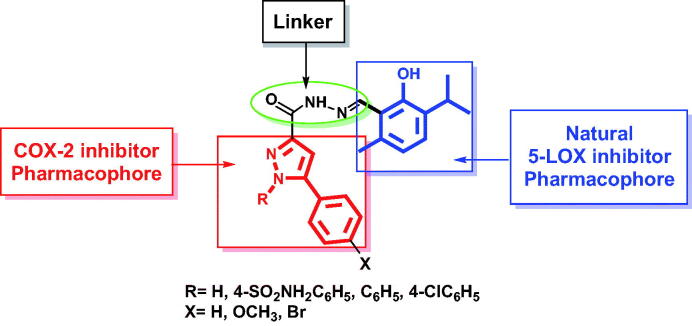
Design of dual COX-2/5-LOX inhibitors.

In the present investigation, the COX-2 inhibitor pharmacophore, 1,5-diarylpyrazole, was hybridised with thymol, a natural 5-LOX inhibitor, through N-acylhydrazone linker in order to simultaneously inhibit of the key inflammatory enzymes COX-2 and 5-LOX to get compounds having *in vivo* anti-inflammatory activity with low side effects and better safety profile (Figure 2).

## Experimental

### Chemistry

Melting points were determined in open glass capillaries using a Griffin melting point apparatus or an electrothermal capillary tube melting point apparatus and are all uncorrected. Infra-red spectra (IR) were recorded, using KBr discs, by a Perkin-Elmer 1430 Infra-red spectrophotometer in the Central Laboratory, Faculty of Pharmacy, Alexandria University, and Schimadzu FT-IR Affinity-1 Spectrometer in Faculty of Pharmacy, Cairo University. Nuclear magnetic resonance (1H-NMR and 13C-NMR) were determined using Bruker High-Performance Digital FT-NMR Spectrometer Avance III 400 MHz, Faculty of Pharmacy, Cairo University in using deuterated dimethylsulphoxide as a solvent. The data were reported as chemical shifts or δ values (ppm) relative to tetramethylsilane (TMS) as internal standard. Signals are indicated by the following abbreviations: s = singlet, d = doublet, t = triplet, q = quartette, and m = multiplet. Electron impact mass spectra (EIMS) were run on a gas chromatograph/mass spectrophotometer at Al-Azhar University (The Regional Centre of Microbiology and Biotechnology). Relative intensity % corresponding to the most characteristic fragments was recorded. Elemental microanalyses were performed at the microanalytical unit, Al-Azhar University (The Regional Centre of Microbiology and Biotechnology). Reaction progress was monitored by thin-layer chromatography (TLC) on silica gel sheets (60 GF254, Merck, Kenilworth, NJ 07033, USA). The spots were visualised by exposure to iodine vapour or UV-lamp at λ 254 nm for few seconds.

Compounds **1a–c**^35^^,^^36^, **2a–c**^37^, **3**^38^^–^^41^ **5a–c**^42–44^, **6a–g,i,**^26^ and **7a–g,i**^45^ were prepared according to reported procedures.

#### General procedure for synthesis of N'-(2-Hydroxy-3-isopropyl-6-methylbenzylidene)-3-(substituted phenyl)-1H-pyrazole-5-carbohydrazides (4a–c)

A mixture of 2-formylthymol **3**, appropriate hydrazides **2a–c**, and few drops of glacial acetic acid in absolute ethanol was refluxed for 4–6 h, cooled to RT, the precipitate was filtered, washed with diethyl ether and recrystallised from ethanol.

#### N'-(2-Hydroxy-3-isopropyl-6-methylbenzylidene)-3-phenyl-1H-pyrazole-5-carbohydrazide; 4a

White crystals, yield 68%, m.p0.164–166 °C. IR (KBr, *v* cm^−1^): 3567.49 (OH); 3266.54 (NH); 1691.30(C=O); 1605.85, 1566.92 (C=N); ^1^H-NMR (400 MHz, DMSO-d_6_δ ppm**):** 1.19(d, 6H, *J=* 6.88 Hz, 2CH_3_ of isopropyl); 2.35(*s*, 3H, CH_3_ of 6-methylthymol); 3.25–3.28 (*m,* 1H, CH of isopropyl); 6.57(*s*, 1H, CH of C4 of pyrazole); 6.76(*d,* 1H, *J=* 7.72 Hz, C5 of thymol); 6.95–7.00(*m,* 3H, C3, C4, C5 of phenyl); 7.24(*d,* 1H, *J=* 7.72 Hz, C4 of thymol); 7.40 (*d,* 2H, *J=* 7.6 Hz, C2, C6 of phenyl); 9.27(br.*s*, 1H, CH of CH=N); 12.47(*s*, 1H, OH, D_2_O exchangeable); 12.60(*s*, 1H, NH of NH-N=, D_2_O exchangeable); 13.87(br.*s*, 1H of NH of pyrazole, D_2_O exchangeable); ^13^C-NMR (100 MHz, DMSO-d_6_δ ppm): 19.26 (CH_3_ of 6-methylthymol); 22.86 (2CH_3_ of isopropyl); 26.61 (CH of isopropyl); 115.33 (C4 of pyrazole); 116.83 (C1 of thymol); 120.85 (C5 of thymol); 121.65 (C3 of thymol); 125.60 (C4 of thymol); 130.39 (C4 of phenyl); 132.94 (C3 and C5 of phenyl); 133.31 (C2 and C6 of phenyl); 133.79 (C1 of phenyl); 138.35 (C6 of thymol); 141.75 (C3 of pyrazole); 155.25 (C5 of pyrazole); 157.91 (CH of CH = N); 163.67 (C2 of thymol); 164.91 (C = O); EIMS, m/z (relative abundance %): 363.01(M^+.^+1) (8.3), 362.17 (M^+.^) (3.04), 187.06 (100.00); 171.28 (74.13); Analysis Calcd for C_21_H_22_N_4_O_2_ (362.17): C, 69.59; H, 6.12; N, 15.46. Found: C, 69.26; H, 5.88; N, 15.09.

#### 3-(4-Bromophenyl)-N'-(2-hydroxy-3-isopropyl-6-methylbenzylidene)-1H-pyrazole-5-carbohydrazide; 4b

Camel crystals, yield 75%, m.p0.260–262 °C. IR (KBr, *v* cm^−1^): 3444.15 (OH); 3228.94 (NH); 1666.98(C = O); 1614.04, 1599.59 (C = N); ^1^H-NMR (400 MHz, DMSO-d_6_δ ppm): 1.19(*d,* 6H, *J=* 6.84 Hz, 2CH_3_ of isopropyl); 2.38(*s*, 3H, CH_3_ of 6-methylthymol); 3.27–3.31(*m,* 1H, CH of isopropyl); 6.71(*d,* 1H, *J=* 7.60 Hz, C5 of thymol); 7.13(*d,* 1H, *J=* 7.60 Hz, C4 of thymol); 7.32(*s*, 1H, CH of C4 of pyrazole); 7.70(*d,* 2H, *J=* 7.68 Hz, C3, C5 of bromophenyl); 7.82(*d,* 2H, *J=* 7.68 Hz, C2, C6 of bromophenyl); 9.06(br.*s*, 1H, CH of CH=N); 12.17(*s*, 1H, OH, D_2_O exchangeable); 12.61(*s*, 1H, NH of NH-N=, D_2_O exchangeable); 13.93(br.*s*, 1H of NH of pyrazole, D_2_O exchangeable); Analysis Calcd for C_21_H_21_BrN_4_O_2_ (441.33): C, 57.15; H, 4.80; N, 12.70. Found: C, 56.71; H, 4.64; N, 12.85.

#### N'-(2-Hydroxy-3-isopropyl-6-methylbenzylidene)-3–(4-methoxy phenyl)-1H-pyrazole-5-carbohydrazide; 4c

Linen crystals, yield 65%, m.p. 246–248 °C. IR (KBr, *v* cm^−1^): 3421.72(OH); 3245.12(NH); 1668.28(C = O); 1609.13, 1600.14(C = N); ^1^H-NMR (400 MHz, DMSO-d_6_δ ppm): 1.19(*d,* 6H, *J =* 6.88 Hz, 2CH_3_ of isopropyl); 2.37(*s*, 3H, CH_3_ of 6-methylthymol); 3.27–3.31(*m,* 1H, CH of isopropyl); 3.82(s, 3H, CH_3_ of OCH_3_); 6.70(*d,* 1H, *J=* 7.60 Hz, C5 of thymol); 7.06(*d,* 2H, *J=* 8.40 Hz, C3, C5 of p-methoxyphenyl); 7.11–7.15(*m,* 2H, C4 of thymol, CH of C4 of pyrazole); 7.80(*d,* 2H, *J=* 8.40 Hz, C2, C6 of p-methoxyphenyl); 9.07(br.*s,* 1H, CH of CH=N); 12.11(*s,* 1H, OH, D_2_O exchangeable); 12.64(*s,* 1H, NH of NH-N=, D_2_O exchangeable); 13.70(*s,* 1H of NH of pyrazole, D_2_O exchangeable); ^13^C-NMR (100 MHz, DMSO-d_6_δ ppm): 19.12(CH_3_ of 6-methylthymol); 22.78(2CH_3_ of isopropyl); 26.49(CH of isopropyl); 55.74(CH_3_ of OCH_3_); 103.14(C4 of pyrazole); 114.94(2 C, C3, C5 of p-methoxyphenyl); 115.80(C1 of thymol); 121.23(C5 of thymol); 121.61(C1 of p-methoxyphenyl); 127.43(2 C, C2, C6 of p-methoxyphenyl); 128.23(C4 of thymol); 133.69(C3 of thymol); 136.01(C6 of thymol); 144.30(C3 of pyrazole); 146.53(C5 of pyrazole); 149.01(CH of CH = N); 156.42(C2 of thymol); 158.28(C = O); 160.01(C4 of p-methoxyphenyl**);** EIMS, m/z (relative abundance %): 393 (M^+.^+1) (13.58); 392 (M^+.^) (43.87); 217 (100.00); 216 (51.88); 201 (73.85); 200 (66.89); Analysis Calcd for C_22_H_24_N_4_O_3_ (392.46): C, 67.33; H, 6.16; N, 14.28. Found: C, 66.98; H, 6.45; N, 14.57.

#### General procedure for synthesis of Methyl 5-(4-substitutedphenyl)-1-(4-substitutedphenyl)-1H-pyrazole-3-carboxylates (6a–i)

A mixture of sodium salt of diketone **1a–c** (1 mol) and substituted phenylhydrazine hydrochloride **5a–c** (1 mol) in glacial acetic acid (30 ml) was refluxed for 24 h, then cooled to RT and poured on crushed ice, filtered, washed with water, air-dried and then washed with diethyl ether and recrystallised from methanol.

#### Methyl 5-(4-bromophenyl)-1-(4-chlorophenyl)-1H-pyrazole-3-carboxylate; 6h

Pale pink crystals, yield 80%, m.p0.96–98 °C. IR (KBr, *v* cm^−1^): 1749.15(C = O); 1587.70(C = N); ^1^H-NMR (400 MHz, DMSO-d_6_δ ppm): 3.87(*s,* 3H, CH_3_ of OCH_3_); 7.20(*s,* 1H, CH of C4 of pyrazole); 7.33(*d,* 2H, *J=* 8.84 Hz, C3, C5 of chlorophenyl); 7.53–7.56(*m,* 4H, C2, C6 of chlorophenyl, C3, C5 of bromophenyl); 7.74(*d,* 2H, *J=* 8.36 Hz, C2, C6 of bromophenyl); EIMS, m/z (relative abundance %): 394 (M^+.^+2) (28.07); 393 (M^+.^+1) (11.87); 392(M^+.^) (70.52); 391 (9.85); 129(21.55); 127 (91.87); 126 (17.15); 75(37.57); 74(34.46); 73(26.25); 55(52.76); 57(59.94); 43(100); 42(32.85); 41(30.96); Analysis Calcd for C_17_H_12_BrClN_2_O_2_ (391.65): C, 52.14; H, 3.09; N, 7.15. Found: C, 51.98; H, 3.34; N, 7.28.

#### General procedure for synthesis of 5-(4-Substitutedphenyl)-1-(4-substitutedphenyl)-1H-pyrazole-3-carbohydrazides (7a–i)

To methyl pyrazole-3-carboxylate **6a–i** (1 mol) in ethanol (20–25 ml), hydrazine hydrate (5 mol) was added and the reaction mixture was refluxed for 7–9 h, then the solvent was evaporated under reduced pressure, then the precipitate was washed with cold water, then with ether, air-dried and recrystallised from ethanol.

#### 5-(4-Bromophenyl)-1–(4-chlorophenyl)-1H-pyrazole-3-carbohydrazide; 7h

White crystals, yield 74%, m.p0.86–88 °C. IR (KBr, *v* cm^−1^): 3375.87, 3250.12(NH, NH_2_); 1755.55(C = O); 1571.75(C = N); ^1^H-NMR (400 MHz, DMSO-d_6_δ ppm): 4.49(*s,* 2H, NH_2_, D_2_O exchangeable); 7.01 (*s,* 1H, CH of C4 of pyrazole); 7.26–7.28(*m,* 2H, C3, C5 of chlorophenyl); 7.35(*d,* 2H, *J=* 8.76 Hz, C3, C5 of bromophenyl); 7.38–7.40(*m,* 2H, C2, C6 of chlorophenyl); 7.52(*d,* 2H, *J=* 8.76 Hz, C2, C6 of bromophenyl); 9.61(*s,* 1H, NH, D_2_O exchange- able); EIMS, m/z (relative abundance %): 394 (M^+.^+2) (19.90); 393 (M^+.^+1) (78.76); 392(M^+.^) (16.23); 391 (60.63); 363(27.69); 362(13.58); 361(100); 360(14.11); 359(41.32); 75(66.24); 111(50.79); Analysis Calcd for C_16_H_12_BrClN_4_O (391.65): C, 49.07; H, 3.09; N, 14.31. Found: C, 49.38; H, 3.23; N, 14.50.

#### General procedure for synthesis of 5–(4-Substitutedphenyl)-1-(4-substitutedphenyl)-N'-(2-hydroxy-3-isopropyl-6-methylbenzylidene)-1H-pyrazole-3-carbohydrazides (8a–i)

Equimolar amount of 2-formylthymol **3** and appropriate hydrazide **7a–i** in absolute ethanol and few drops of glacial acetic acid were refluxed for 4–6 h, cooled to RT, the precipitate was filtered, washed with diethyl ether, and recrystallised from ethanol.

#### 4-(3-(2–(2-Hydroxy-3-isopropyl-6-methylbenzylidene)hydrazine-1-carbonyl)-5-phenyl-1H-pyrazol-1-yl)benzenesulfonamide; 8a

White crystals, yield 79%, m.p0.220–222 °C. IR (KBr, *v* cm^−1^): 3568.31 (OH); 3272.15 (NH); 1662.64(C = O); 1597.06(C = N); ^1^H-NMR (400 MHz, DMSO-d_6_δ ppm): 1.19(*d,* 6H, *J=* 6.80 Hz, 2CH_3_ of isopropyl); 2.37(*s,* 3H, CH_3_ of 6-methylthymol); 3.1–3.2(*m,* 1H, CH of isopropyl); 6.71(*d,* 1H, *J=* 7.68 Hz, C5 of thymol); 7.14(*d,* 1H, *J=* 7.68 Hz, C4 of thymol); 7.24(*s,* 1H, CH of C4 of pyrazole); 7.35–7.36(*m,* 2H, C2, C6 of phenyl); 7.42–7.43(*m,* 3H, C3, C4, C5 of phenyl); 7.52(*s,* 2H of NH_2_ of sulfamoyl, D_2_O exchangeable); 7.61(*d,* 2H, *J=* 8.36 Hz, C3, C5 of sulfamoylphenyl); 7.61(*d,* 2H, *J=* 8.36 Hz, C2, C6 of sulfamoylphenyl); 9.07(*s,* 1H, CH of CH=N); 12.25(*s,* 1H, OH, D_2_O exchangeable); 12.58(*s,* 1H, NH of NH-N=, D_2_O exchangeable); ^13^C-NMR (100 MHz, DMSO-d_6_δ ppm): 19.13(CH_3_ of 6-methylthymol); 22.77(2CH_3_ of isopropyl); 26.50(CH of isopropyl); 109.42(C4 of pyrazole); 115.69(C1 of thymol); 121.30(C5 of thymol); 125.45(2C, C2, C6 of sulfamoylphenyl); 127.21(2C, C3, C5 of sulfamoylphenyl); 128.46(C4 of thymol); 129.22(2C, C3, C5 of phenyl); 129.26(C4 of phenyl); 129.32(2C, C2, C6 of phenyl);129.62(C1 of phenyl); 133.75(C3 of thymol); 136.16(C6 of thymol); 141.97(C4 of sulfamoylphenyl); 144.35(C3 of pyrazole); 145.40(C1 of sulfamoylphenyl); 146.54(C5 of pyrazole); 149.68(CH of CH = N); 156.49(C2 of thymol); 157.43(C = O); EIMS, m/z (relative abundance %): 518 (M^+.^+1) (14.15); 517 (M^+.^) (36.90); 326 (88.11); 160 (100.00); 77 (65.57); Analysis Calcd for C_27_H_27_N_5_O_4_S (517.60): C, 62.65; H, 5.26; N, 13.53; S, 6.19. Found: C, 62.84; H, 5.38; N, 13.79; S, 6.27.

#### 4-(5-(4-Bromophenyl)-3-(2-(2-hydroxy-3-isopropyl-6-methylbenzylidene)hydrazine-1-carbonyl)-1H-pyrazol-1-yl)benzene sulphonamide; 8b

White crystals, yield 80%, m.p0.253–255 °C. IR (KBr, *v* cm^−1^): 3545.16(OH); 3263.56(NH); 1689.64(C = O); 1593.20(C = N); ^1^H-NMR (400 MHz, DMSO-d_6_δ ppm): 1.19(*d,* 6H, *J=* 6.84 Hz, 2CH_3_ of isopropyl); 2.37(*s,* 3H, CH_3_ of 6-methylthymol); 3.27–3.31(*m,* 1H, CH of isopropyl); 6.71(*d,* 1H, *J=* 7.68 Hz, C5 of thymol); 7.14(*d,* 1H, *J=* 7.68 Hz, C4 of thymol); 7.28–7.31(*m,* 3H, CH of C4 of pyrazole, C3, C5 of bromophenyl); 7.52(*s,* 2H of NH_2_ of sulfamoyl, D_2_O exchangeable); 7.62–7.65(*m,* 4H, C2, C6 of bromophenyl C3, C5 of sulfamoylphenyl); 7.93(*d,* 2H, *J=* 8.48 Hz, C2, C6 of sulfamoylphenyl); 9.06(*s,* 1H, CH of CH=N); 12.26(*s,* 1H, OH, D_2_O exchangeable); 12.57(*s,* 1H, NH of NH-N=, D_2_O exchangeable); ^13^C-NMR (100 MHz, DMSO-d_6_δ ppm): 19.13(CH_3_ of 6-methylthymol); 22.78(2CH_3_ of isopropyl); 26.50(CH of isopropyl); 109.71(C4 of pyrazole); 115.67(C1 of thymol); 121.31(C5 of thymol); 123.21(C4 of bromophenyl); 126.63(2C, C2, C6 of sulfamoylphenyl); 127.31(2C, C3, C5 of sulfamoylphenyl); 128.43(C1 of bromophenyl); 128.49(C4 of thymol); 131.31(2C, C2, C6 of bromophenyl); 132.30(2C, C3, C5 of bromophenyl); 133.75(C3 of thymol); 136.17(C6 of thymol); 141.73(C4 of sulfamoylphenyl); 144.25(C3 of pyrazole); 144.44(C1 of sulfamoylphenyl); 146.59(C5 of pyrazole); 149.74(CH of CH=N); 156.48(C2 of thymol); 157.33(C = O); EIMS, m/z (relative abundance %): 599 (M^+.^+2)(6.71); 598 (M^+.^+1) (4.23); 597 (M^+.^) (16.77); 423 (68.60); 421 (64.12); 420 (100.00); 406 (77.05); 404 (72.95); Analysis Calcd for C_27_H_26_ BrN_5_O_4_S (596.50): C, 54.37; H, 4.39; N, 11.74; S, 5.37. Found: C, 54.66; H, 4.60; N, 11.96; S, 5.51.

#### 4-(3-(2-(2-Hydroxy-3-isopropyl-6-methylbenzylidene)hydrazine-1-carbonyl)-5-(4-methoxyphenyl)-1H-pyrazol-1-yl)benzenesulfonamide; 8c

Pale white crystals, yield 77%, m.p. 245–247 °C. IR (KBr, *v* cm^−1^): 3567.41 (OH); 3266.82 (NH); 1689.64 (C = O); 1597.06(C = N); ^1^H-NMR (400 MHz, DMSO-d_6_δ ppm): 1.12(*d,* 6H, *J=* 6.76 Hz, 2CH_3_ of isopropyl); 2.37(*s,* 3H, CH_3_ of 6-methylthymol); 3.27–3.31(*m,* 1H, CH of isopropyl); 3.78(*s,* 3H, CH_3_ of OCH_3_); 6.71(*d,* 1H, *J=* 7.72 Hz, C5 of thymol); 6.98(*d,* 2H, *J=* 8.72 Hz, C3, C5 of p-methoxyphenyl); 7.14(*d,* 1H, *J=* 7.72 Hz, C4 of thymol); 7.15(*s,* 1H, CH of C4 of pyrazole); 7.28(*d,* 2H, *J=* 8.72 Hz, C2, C6 of p-methoxyphenyl); 7.52(*s,* 2H of NH_2_ of sulfamoyl, D_2_O exchangeable); 7.61(*d,* 2H, *J=* 8.52 Hz, C3, C5 of sulfamoylphenyl); 7.91(*d,* 2H, *J=* 8.52 Hz, C2, C6 of sulfamoylphenyl); 9.06(*s,* 1H, CH of CH=N); 12.22(*s,* 1H, OH, D_2_O exchangeable); 12.58(*s,* 1H, NH of NH-N=, D_2_O exchangeable); ^13^C-NMR (100 MHz, DMSO-d_6_δ ppm): 19.13(CH_3_ of 5-methyl thymol); 22.78(2CH_3_ of isopropyl); 26.50(CH of isopropyl); 55.73(CH_3_ of OCH_3_); 108.88(C4 of pyrazole); 114.78(2C, C3, C5 of p-methoxyphenyl); 115.70(C1 of thymol); 121.30(C5 of thymol); 121.39 (C1 of p-methoxyphenyl); 126.55(2C, C2, C6 of sulfamoyl phenyl); 127.20(2C, C3, C5 of sulfamoylphenyl); 128.45(C4 of thymol); 130.67(2C, C2, C6 of p-methoxyphenyl); 133.75(C3 of thymol); 136.15(C6 of thymol); 142.11 (C4 of sulfamoylphenyl); 144.23(C3 of pyrazole); 145.30(C1 of sulfamoyl phenyl); 146.43 (C5 of pyrazole); 149.62(CH of CH = N); 156.47(C2 of thymol); 157.49(C = O); 160.25(C4 of p-methoxyphenyl); EIMS, m/z (relative abundance %): 549 (M^+.^+1) (38.26); 548 (M^+.^) (63.25); 521 (72.07); 404 (78.65); 241 (70.30); 163 (100.00); 135 (63.36); Analysis Calcd for C_28_H_29_N_5_O_5_S (547.63): C, 61.41; H, 5.34; N, 12.79; S, 5.85. Found: C, 61.30; H, 5.56; N, 13.06; S, 5.97.

#### N'-(2-Hydroxy-3-isopropyl-6-methylbenzylidene)-1,5-diphenyl-1H-pyrazole-3-carbo-hydrazide; 8d

White crystals, yield 75%, m.p. 232–235 °C. IR (KBr, *v* cm^−1^): 3421.72 (OH); 3270.97 (NH); 1651.07(C = O); 1597.06(C = N); ^1^H-NMR (400 MHz, DMSO-d_6_δ ppm): 1.19(*d,* 6H, *J=* 6.88 Hz, 2CH_3_ of isopropyl); 2.36(*s,* 3H, CH_3_ of 6-methylthymol); 3.27–3.31(*m,* 1H, CH of isopropyl); 6.70(*d,* 1H, *J=* 7.72 Hz, C5 of thymol); 7.13(*d,* 1H, *J=* 7.72 Hz, C4 of thymol); 7.21(*s,* 1H, CH of C4 of pyrazole); 7.31–7.32(*m,* 2H, C2, C6 of phenyl); 7.37–7.38(*m,* 3H, C3, C4, C5 of phenyl); 7.41–7.43(*m,* 2H, C2, C6 of N-phenyl); 7.48–7.50(*m,* 3H, C3, C4, C5 of N-phenyl); 9.08(*s,* 1H, CH of CH=N); 12.22(*s,* 1H, OH, D_2_O exchangeable); 12.60(*s,* 1H, NH of NH-N=, D_2_O exchangeable); ^13^C-NMR (100 MHz, DMSO-d_6_δ ppm): 19.11(CH_3_ of 6-methylthymol); 22.78(2CH_3_ of isopropyl); 26.50(CH of isopropyl); 108.73(C4 of pyrazole); 115.73(C1 of thymol); 121.27(C5 of thymol); 126.48(2C, C2, C6 of N-phenyl); 128.39(C4 of thymol); 129.09(2C, C2, C6 of phenyl); 129.13(3C, C3, C5 of phenyl, C4 of N-phenyl); 129.29(C4 of phenyl); 129.46(C1 phenyl); 129.72(2C, C3, C5 of N-phenyl); 133.72(C3 of thymol); 136.12(C6 of thymol); 139.71(C1 of N-phenyl); 145.16(C3 of pyrazole); 145.91(C5 of pyrazole); 149.56(CH of CH = N); 156.46(C2 of thymol); 157.62(C = O); EIMS, m/z (relative abundance %): 439 (M^+.^+1) (20.10); 438 (M^+.^) (45.95); 247 (100.00); 245 (33.97); Analysis Calcd for C_27_H_26_N_4_O_2_ (438.53): C, 73.95; H, 5.98; N, 12.78. Found: C, 74.23; H, 5.87; N, 13.14.

#### 5-(4-Bromophenyl)-N'-(2-hydroxy-3-isopropyl-6-methylbenzylidene)-1-phenyl-1H-pyrazole-3-carbohydrazide; 8e

White crystals, yield 82%, m.p. 0.209–211 °C. IR (KBr, *v* cm^−1^): 3545.16(OH); 3278.69(NH); 1651.07(C = O); 1597.06(C = N); ^1^H-NMR (400 MHz, DMSO-d_6_δ ppm): 1.20(*d,* 6H, *J=* 6.88 Hz, 2CH_3_ of isopropyl); 2.36(*s,* 3H, CH_3_ of 6-methylthymol); 3.27–3.31(*m,* 1H, CH of isopropyl); 6.71(*d,* 1H, *J=* 7.72 Hz, C5 of thymol); 7.14(*d,* 1H, *J=* 7.72 Hz, C4 of thymol) ; 7.23(*s,* 1H, CH of C4 of pyrazole); 7.27(*d,* 2H, *J=* 8.00 Hz, C3, C5 of p-bromophenyl); 7.43–7.45(*m,* 2H, C2, C6 of N-phenyl); 7.51–7.53(*m,* 3H, C3, C4, C5 of N-phenyl); 7.59(*d,* 2H, *J=* 8.00 Hz, C2, C6 of p-bromophenyl); 9.07(*s,* 1H, CH of CH=N); 12.24(*s,* 1H, OH, D_2_O exchangeable); 12.59(*s,* 1H, NH of NH-N=, D_2_O exchangeable); EIMS, m/z (relative abundance %): 519 (M^+.^+2)(41.16); 518 (M^+.^+1) (62.23); 517 (M^+.^) (19.71); 516 (67.32); 341 (82.53); 327 (100.00); 325 (70.56); 44 (88.69); Analysis Calcd for C_27_H_25_BrN_4_O_2_ (517.43): C, 62.67; H, 4.87; N, 10.83. Found: C, 62.50; H, 4.98; N, 11.15.

#### 5-(4-Methoxyphenyl)-N'-(2-hydroxy-3-isopropyl-6-methylbenzylidene)-1-phenyl-1H-pyrazole-3-carbohydrazide; 8f

White crystals, yield 75%, m.p. 0.227–229 °C. IR (KBr, *v* cm^−1^): 3448.72 (OH); 3279.99(NH); 1685.79(C = O); 1597.06(C = N); ^1^H-NMR (400 MHz, DMSO-d_6_δ ppm): 1.19(*d,* 6H, *J=* 6.88 Hz, 2CH_3_ of isopropyl); 2.35(*s,* 3H, CH_3_ of 6-methylthymol); 3.27–3.31(*m,* 1H, CH of isopropyl); 3.76(*s,* 3H, CH_3_ of OCH_3_); 6.70(*d,* 1H, *J=* 7.72 Hz, C5 of thymol); 6.91(*d,* 2H, *J=* 8.60 Hz, C3, C5 of p-methoxyphenyl); 7.12–7.14(*m,* 2H, C4 of thymol, CH of C4 of pyrazole) ; 7.23(*d,* 2H, *J=* 8.60 Hz, C2, C6 of p-methoxyphenyl); 7.41–7.43(*m,* 2H, C2, C6 of N-phenyl); 7.48–7.50(*m,* 3H, C3, C4, C5 of N-phenyl); 9.07(*s,* 1H, CH of CH=N); 12.19(*s,* 1H, OH, D_2_O exchangeable); 12.61(*s,* 1H, NH of NH-N=, D_2_O exchangeable); ^13^C-NMR (100 MHz, DMSO-d_6_δ ppm): 19.10(CH_3_ of 6-methylthymol); 22.78(2CH_3_ of isopropyl); 26.50(CH of isopropyl); 55.66(CH_3_ of OCH_3_); 108.14(C4 of pyrazole); 114.58(2C, C3, C5 of p-methoxyphenyl); 115.72(C1 of thymol); 121.26(C5 of thymol); 121.68(C1 of p-methoxyphenyl); 126.47(2C, C2, C6 of N-phenyl); 128.36(C4 of thymol); 129.20(C4 of N-phenyl); 129.71(2C, C3, C5 of N-phenyl); 130.47(2C, C2, C6 of p-methoxyphenyl); 133.72(C3 of thymol); 136.10(C6 of thymol); 139.84(C1 of N-phenyl); 145.06(C3 of pyrazole); 145.81(C5 of pyrazole); 149.49(CH of CH = N); 156.46(C2 of thymol); 157.69(C = O); 160.01(C4 of p-methoxyphenyl); EIMS, m/z (relative abundance %): 469 (M^+.^+1) (39.25); 468 (M^+.^) (75.20); 467(19.50); 293(100.00); 277 (95.75); 276 (37.40).

Analysis Calcd for C_28_H_28_N_4_O_3_ (468.56): C, 71.78; H, 6.02; N, 11.96. Found: C, 72.11; H, 6.24; N, 11.87.

#### 1-(4-Chlorophenyl)-N'-(2-hydroxy-3-isopropyl-6-methylbenzylidene)-5-phenyl-1H-pyrazole-3-carbohydrazide; 8g

White crystals, yield 86%, m.p. 0.245–247 °C. IR (KBr, *v* cm^−1^): 3545.15 (OH); 3266.25(NH); 1738.54 (C = O); 1586.19 1570.32(C = N); ^1^H-NMR (400 MHz, DMSO-d_6_δ ppm): 1.19(*d,* 6H, *J=* 6.82 Hz, 2CH_3_ of isopropyl); 2.35(*s,* 3H, CH_3_ of 6-methylthymol); 3.27–3.31(*m,* 1H, CH of isopropyl); 6.72(*d,* 1H, *J=* 7.72 Hz, C5 of thymol); 7.14 (*d,* 1H, *J=* 7.72 Hz, C4 of thymol); 7.21(*s,* 1H, CH of C4 of pyrazole); 7.32–7.34(*m,* 2H, C3, C5 of phenyl); 7.40–7.42(*m,* 3H, C2, C4, C6 of phenyl); 7.46 (*d,* 2H, *J=* 8.62 Hz, C3, C5 of chlorophenyl); 7.57 (*d,* 2H, *J=* 8.62 Hz, C2, C6 of chlorophenyl); 9.08(*s,* 1H, CH of CH=N); 12.24(*s,* 1H, OH, D_2_O exchangeable); 12.58(*s,* 1H, NH of NH-N=, D_2_O exchangeable); Analysis Calcd for C_26_H_23_ClN_4_O_2_ (458.95): C, 68.04; H, 5.05; N, 12.21. Found: C, 68.19; H, 5.27; N, 12.09.

#### 5-(4-Bromophenyl)-1-(4-chlorophenyl)-N'-(2-hydroxy-3-isopropyl-6-methylbenzylidene)-1H-pyrazole-3-carbohydrazide; 8h

White crystals, yield 70%, m.p. 0.255–257 °C. IR (KBr, *v* cm^−1^): 3448.72 (OH); 3277.99(NH); 1744.95 (C = O); 1599.59, 1572.07(C = N); ^1^H-NMR (400 MHz, DMSO-d_6_δ ppm): 1.19(*d,* 6H, *J=* 6.88 Hz, 2CH_3_ of isopropyl); 2.36(*s,* 3H, CH_3_ of 6-methylthymol); 3.27–3.31(*m,* 1H, CH of isopropyl); 6.71(*d,* 1H, *J=* 7.72 Hz, C5 of thymol); 7.13(*d,* 1H, *J=* 7.72 Hz, C4 of thymol); 7.25(*s,* 1H, CH of C4 of pyrazole); 7.28 (*d,* 2H, *J=* 8.3 Hz, C3, C5 of chlorophenyl); 7.47(*d,* 2H, *J=* 8.5 Hz, C3, C5 of bromophenyl); 7.58–7.64(*m,* 4H, C2, C6 of chlorophenyl, C2, C6 of bromophenyl); 9.07(*s,* 1H, CH of CH=N); 12.25(*s,* 1H, OH, D_2_O exchangeable); 12.58(*s,* 1H, NH of NH-N=, D_2_O exchangeable); EIMS, m/z (relative abundance %): 555 (M^+.^+4) (11.5); 553 (M^+.^+2)(30.84); 552 (M^+.^+1)(36.91); 551 (M^+.^) (100.00); 548 (58.43); 375 (62.53); Analysis Calcd for C_27_H_24_BrClN_4_O_2_ (551.87): C, 58.76; H, 4.38; N, 10.15. Found: C, 59.02; H, 4.51; N, 10.38.

#### 5-(4-Methoxyphenyl)-1-(4-chlorophenyl)-N'-(2-hydroxy-3-isopropyl-6-methylbenzylidene)-1H-pyrazole-3-carbohydrazide; 8i

White crystals, yield 75%, m.p. 218–220 °C. IR (KBr, *v* cm^−1^): 3417.86 (OH); 3259.69 (NH); 1717.40(C = O); 1604.84, 1594.60 (C = N); ^1^H-NMR (400 MHz, DMSO-d_6_δ ppm): 1.19(*d,* 6H, *J=* 6.88 Hz, 2CH_3_ of isopropyl); 2.36(*s,* 3H, CH_3_ of 6-methylthymol); 3.27–3.31(*m,* 1H, CH of isopropyl); 3.77(*s,* 3H, CH_3_ of OCH_3_); 6.70(*d,* 1H, *J=* 7.72 Hz, C5 of thymol); 6.96(*d,* 2H, *J=* 8.60 Hz, C3, C5 of p-methoxyphenyl); 7.12–7.14(*m,* 2H, C4 of thymol, CH of C4 of pyrazole); 7.24(*d,* 2H, *J=* 8.60 Hz, C2, C6 of p-methoxyphenyl); 7.45(*d,* 2H, *J=* 8.60 Hz, C3, C5 of chlorophenyl); 7.57(*d,* 2H, *J=* 8.60 Hz, C2, C6 of chlorophenyl); 9.07(*s,* 1H, CH of CH=N); 12.21(*s,* 1H, OH, D_2_O exchangeable); 12.60(*s,* 1H, NH of NH-N=, D_2_O exchangeable); ^13^C-NMR (100 MHz, DMSO-d_6_δ ppm): 19.12(CH_3_ of 6-methylthymol); 22.77(2CH_3_ of isopropyl); 26.50(CH of isopropyl); 55.68(CH_3_ of OCH_3_); 108.45(C4 of pyrazole); 114.69(2C, C3, C5 of p-methoxyphenyl); 115.72(C1 of thymol); 121.27(C5 of thymol); 121.44(C1 of p-methoxyphenyl); 128.07(2C, C2, C6 of chlorophenyl); 128.39(C4 of thymol); 129.73(2C, C2, C6 of p-methoxyphenyl); 130.58(2C, C3, C5 of chlorophenyl); 133.55(C4 of chlorophenyl); 133.73(C3 of thymol); 136.12(C6 of thymol); 138.62(C1 of chlorophenyl); 145.19(C3 of pyrazole); 146.11(C5 of pyrazole); 149.56(CH of CH = N); 156.47(C2 of thymol); 157.57(C = O); 160.13(C4 of p-methoxyphenyl); EIMS, m/z (relative abundance %): 503 (M^+.^+1) (40.05); 502 (M^+.^) (100.00); 327 (77.20); 311 (37.85); Analysis Calcd for C_28_H_27_ ClN_4_O_3_ (503.00): C, 66.86; H, 5.41; N, 11.14. Found: C, 67.14; H, 5.56; N, 11.41.

### Biological screening

#### *In vitro* COX-1 and COX-2 inhibitory assay

Compounds **4a–c** and **8a–i** were screened for their ability to inhibit COX-1 and COX-2 enzymes *in vitro*. This was carried out using Cayman colorimetric COX (ovine) inhibitor screening assay kit (Catalog No. 560131) supplied by Cayman chemicals, Ann Arbour, MI according to reported method^46^ (Page: S20, Supplementary file).

#### *In vitro* 5-LOX inhibitory assay

The newly synthesised compounds were screened for their ability to inhibit 5-LOX enzymes. This was carried out using Abnova 5-LOX inhibitor screening assay kit (Catalog No. 760700) according to reported method^47^ (Page: S21, Supplementary file).

#### *In vivo* anti-inflammatory activity

##### Formalin-induced paw oedema

Compounds that showed *in vitro* selectivity indices higher or nearly equivalent to reference drugs towards COX 2 enzyme, were further evaluated for their *in vivo* anti-inflammatory activity applying the formalin-induced paw oedema screening protocol as an acute inflammation model using celecoxib and Diclofenac sodium as reference drugs according to reported procedures^48^^,^^49^ (Approved by AlexU-IACUC)^50^ (Page: S22–23, Supplementary file).

##### Gastric ulcerogenic activity

Compounds were evaluated for acute gastric ulcerogenic effect in adult female Wistar rats. Gross examination was performed for any evidence of hyperaemia, haemorrhage, definite haemorrhagic erosion, or ulcer according to reported procedures^49^^,^^51^ (Approved by AlexU-IACUC)^50^ (Page: S24, Supplementary file).

##### Molecular modelling

The molecular modelling studies were performed using the Molecular Operating Environment (MOE 2016.08) software (Chemical Computing Group, Montreal, Canada).^52^ and the crystal structures of the proteins were downloaded from the Protein Data Bank (PDB) website (Page: S24, Supplementary file).

## Results and discussion

### Chemistry

**Scheme 1.**
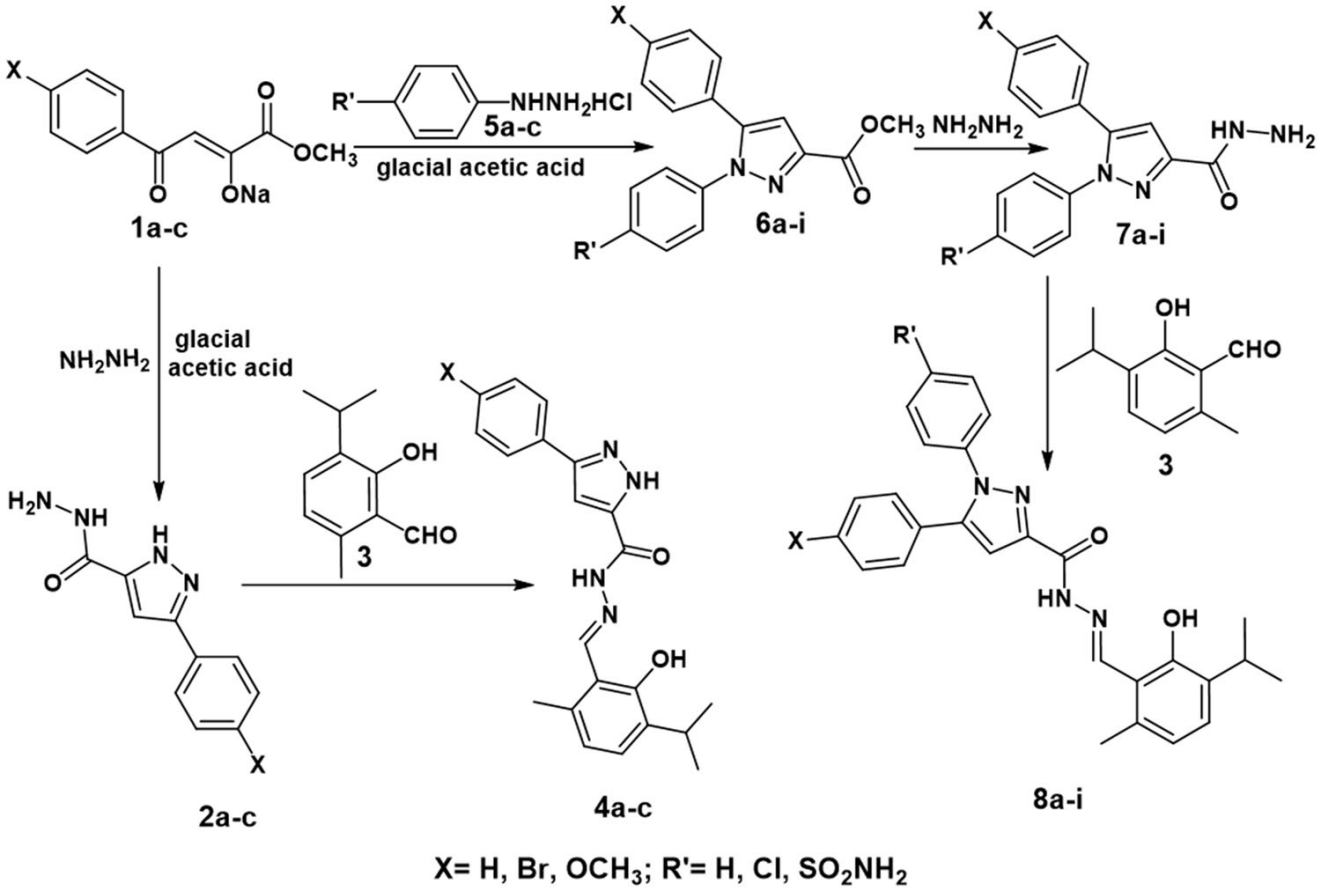
Synthesis of the target thymol–1,5-disubstitutedpyrazole hybrids.

Scheme 1 illustrated the synthesis of the target thymol–1,5-disubstitutedpyrazole hybrids. The dioxobutanoate derivatives^53^ **1a–c** were cyclised using reaction conditions used in Knorr pyrazole synthesis^54^ and celecoxib synthesis^55^ by reaction with either hydrazine hydrate or substituted phenylhydrazine hydrochloride in glacial acetic acid. Hydrazine hydrate resulted in both cyclisation and formation of the hydrazide derivatives **2a–c**. On the other hand, substituted phenylhydrazine hydrochlorides produced only the cyclised methyl esters **6a–i** which were further reacted with hydrazine hydrate to produce the corresponding hydrazides **7a–i**. The hydrazides **2a–c** and **7a–i** were condensed with 2-formylthymol^38–41^ **3** to yield the target thymol-1,5-disubstitutedpyrazole hybrids **4a–c** and **8a–i**, respectively. IR spectra of compounds **4a–c** showed absence of bands assigned to CHO and NH_2_ and presence of absorption bands assigned to C = N, as well as to OH. ^1^H-NMR spectra of compounds **4a–c** were characterised by the absence of signals assigned to NH_2_ protons at their previously recorded positions, and presence of 2D_2_O exchangeable singlets of (NH) functional groups one for (CO-NH-N=) and the other for pyrazole at (12.61–12.64) ppm, (13.70–13.93) ppm, respectively. ^13^C-NMR spectrum of compounds **4a–c** showed the presence of signals of (C = O) and (C3, C4, C5 of pyrazole) at expected chemical shifts. In addition, MS for **4c** showed the molecular ion peak (M^+.^) at m/z 392. On the other hand, IR spectra of compounds **8a–i** were characterised by the absence of absorption bands assigned to (NH_2_ of pyrazole) and (CHO) group and presence of absorption bands assigned to (NH), (C = N) and (C = O) groups as well as (OH) of thymol at their expected absorption regions. ^1^H-NMR spectra of compounds **8a–i** were characterised by the absence of signals assigned to NH_2_ protons at their previously recorded positions, and presence of (CH) proton of C4 of pyrazole at (7.15–7.24) ppm as singlet signal, as well as D_2_O exchangeable singlet of NH proton at 12.57–12.60 ppm. ^13^C-NMR spectra of compounds **8a–i** showed the presence of signals of (C3, C4, C5 of pyrazoles) and (C = N of thiazolidinone) at expected chemical shifts. Besides, MS of compounds **8a-i** showed the molecular ion peak (M^+.^), for **8a** at m/z 517, at 597 for **8b**, at 548 for **8c**, at 438 for **8d**, at 517 for **8e**, at 468 for **8f**, at 551 for **8h,** and at 502 for **8i** (Scheme 1).

### Biological screening

#### *In vitro* COX-1 and COX-2 inhibitory assay

Compounds **4a–c** and **8a–i** were screened for their *in vitro* inhibitory activity against COX-1 and COX-2 enzymes using Cayman colorimetric COX (ovine) inhibitor screening assay kit (Catalog No. 560131) supplied by Cayman chemicals, Ann Arbour, MI^46^. The half-maximal inhibitor concentrations (IC_50_µM) were determined and the selectivity index (SI) values were calculated as IC_50_ (COX-1)/IC_50_ (COX-2) and recorded in Table 1. All compounds showed high activity, in nanomolar range, against COX-2 and high SI to COX-2. Compounds **8b**, **8g**, **8c,** and **4a** displayed nearly equal activity to celecoxib with high SI (316, 268, 204, and 151, respectively) (Table 1).

**Table 1.** *In vitro* COX-1, COX-2, and 5-LOX enzyme inhibitory activities, ^a^IC50 values, and ^b^selectivity indices (SI) of the tested compounds.

Code	COX-1 µM IC_50_	COX-2 µM IC_50_	Selectivity index	5-LOX µM IC_50_
Celecoxib	14.7 ± 0.07	0.045 ± 0.007	327	**–**
Diclofenac sodium	3.9 ± 0.04	0.8 ± 0.009	4.87	**–**
Quercetin	**–**	**–**	**–**	3.34 ± 0.05
**4a**	10.3 ± 0.1	0.068 ± 0.01	151.5	3.05 ± 0.067
**4b**	11.81 ± 0.15	0.17 ± 0.01	69.5	1.84 ± 0.06
**4c**	10.22 ± 0.046	0.19 ± 0.007	53.8	2.91 ± 0.068
**8a**	11.21 ± 0.15	0.12 ± 0.007	93.4	1.96 ± 0.036
**8b**	13.61 ± 0.096	0.043 ± 0.001	316.5	1.58 ± 0.026
**8c**	12.87 ± 0.097	0.063 ± 0.001	204	1.91 ± 0.053
**8d**	10.19 ± 0.036	0.11 ± 0.007	92.6	3.11 ± 0.046
**8e**	11.24 ± 0.075	0.12 ± 0.007	93.6	2.56 ± 0.025
**8f**	11.18 ± 0.57	0.14 ± 0.01	79.8	2.91 ± 0.064
**8g**	12.1 ± 0.16	0.045 ± 0.01	268.8	1.60 ± 0.042
**8h**	12.61 ± 1.36	0.16 ± 0.01	78.8	1.97 ± 0.026
**8i**	12.58 ± 0.46	0.17 ± 0.012	74	2.78 ± 0.016

^a^IC_50_ is the concentration (µM) needed to cause 50% inhibition of COX-1 and COX-2 enzymatic activity. All values are expressed as a mean of three replicates with standard deviation less than 10% of the mean.

^b^Selectivity Index (SI) = (COX-1 IC_50_/COX-2 IC_50_).

#### *In vitro* 5-LOX inhibitory assay

Compounds **4a–c** and **8a–i** were screened for their *in vitro* ability to inhibit 5-LOX enzymes. This was carried out using Abnova 5-LOX inhibitor screening assay kit (Catalog No. 760700)^47^. All compounds showed potent 5-LOX inhibitory activities higher than the reference quercetin. Consequently, compounds **8b**, **8c**, **8g,** and **4a** showed dual inhibitory activities against COX-2 and 5-LOX higher than the references celecoxib and quercetin (Table 1).

#### *In vivo* anti-inflammatory activity

##### Formalin-induced paw oedema test (acute inflammation model)

**Figure 3.**
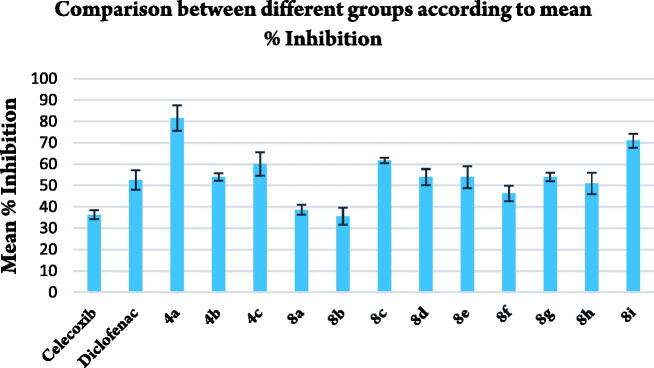
Comparison between different groups according to % inhibition of paw oedema volume.

**Figure 4.**
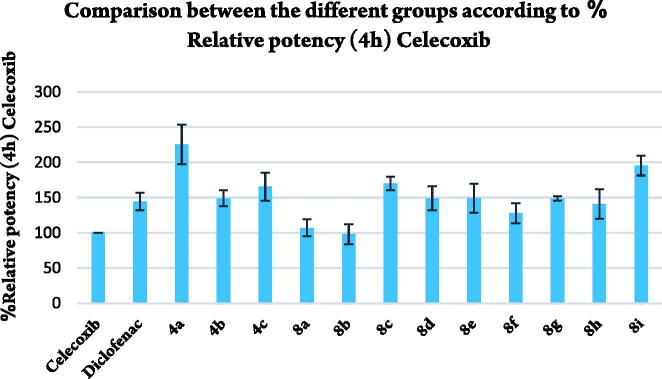
Comparison between the different groups according to % relative potency (4h) celecoxib.

**Figure 5.**
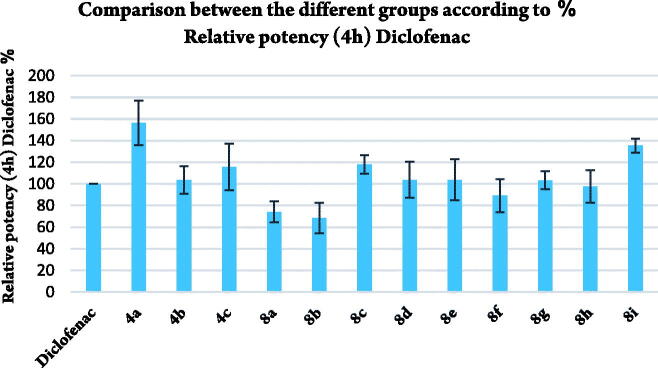
Comparison between the different groups according to %relative potency (4h) diclofenac.

Compounds **4a–c** and **8a–i** were tested for their *in vivo* anti-inflammatory activity using formalin-induced paw oedema test (Acute inflammation model) (Table 2, Figures 3–5). All compounds showed higher % inhibition than celecoxib except **8b** which revealed nearly equal activity to celecoxib. Compounds **4a** and **8i** exhibited double % inhibition exhibited by Celecoxib. All compounds showed higher % inhibition than that of Diclofenac sodium except **8a**, **8b**, **8f,** and **8h** which elicited slightly less % inhibition than that of Diclofenac sodium. Compound **4a** was the most potent with % inhibition 81.93% comparing with 36.37% and 52.37% of celecoxib and diclofenac sodium, respectively.

**Table 2.** *In vivo* anti-inflammatory activities of selected compounds in formalin-induced rat paw oedema bioassay (acute inflammation model).

Code		Volume of oedema (ml)		Abs diff.	% Inhibition of oedema volume	% Relative potency (**4h**) celecoxib	% Relative potency (**4h)** diclofenac
		**0**	**4h**				
**Vehicle DMSO** **(control)**	**1**	4.25	6.47	2.22			
	**2**	4.20	6.37	2.17			
	**3**	4.18	6.31	2.13			
**Mean ± SD**		4.21 ± 0.04	6.38 ± 0.08	2.17 ± 0.05			
**Reference standard (celecoxib)**	**1**	4.25	5.69	1.44	35.10		
	**2**	4.34	5.67	1.33	38.70		
	**3**	4.32	5.70	1.38	35.20		
**Mean ± SD**		4.30 ± 0.05	5.69^a^ ± 0.02	1.38^a^ ± 0.06	36.33 ± 2.05		
**Reference standard (diclofenac)**	**1**	4.20	5.37	1.17	47.30	134.60	
	**2**	4.34	5.33	0.99	54.40	140.50	
	**3**	4.25	5.19	0.94	55.90	158.70	
**Mean ± SD**		4.26 ± 0.07	5.30^ab^ ± 0.09	1.03^ab^ ± 0.12	52.53^b^ ± 4.59	144.6 ± 12.56	
**4a**	**1**	5.15	5.50	0.35	84.20	239.70	178.10
	**2**	5.10	5.65	0.55	74.70	192.90	137.30
	**3**	5.25	5.55	0.30	85.90	244.0	153.80
**Mean ± SD**		5.17^abc^ ± 0.08	5.57^ac^ ± 0.08	0.40^abc^ ± 0.13	81.60^bc^ ± 6.04	225.5^c^ ± 28.34	156.4 ± 20.52
**4b**	**1**	5.20	6.18	0.98	55.90	159.0	118.10
	**2**	5.15	6.17	1.02	53.0	136.90	97.50
	**3**	5.15	6.15	1.0	53.10	150.70	95.0
**Mean ± SD**		5.17^abc^ ± 0.03	6.17^abc^ ± 0.02	1.0^ab^ ± 0.02	54.0^b^ ± 1.65	148.9 ± 11.16	103.5 ± 12.68
**4c**	**1**	5.35	6.10	0.75	66.20	188.50	140.0
	**2**	5.30	6.20	0.90	58.50	151.20	107.60
	**3**	5.25	6.20	0.95	55.40	157.30	99.20
**Mean ± SD**		5.30^abc^ ± 0.05	6.17^abc^ ± 0.06	0.87^ab^ ± 0.10	60.03^b^ ± 5.56	165.7 ± 20.01	115.6 ± 21.54
**8a**	**1**	5.02	6.35	1.33	40.10	114.10	84.80
	**2**	4.96	6.35	1.39	35.90	92.90	66.10
	**3**	5.02	6.30	1.28	39.90	113.30	71.40
**Mean ± SD**		5.0^abc^ ± 0.03	6.33^bc^ ± 0.03	1.33^ac^ ± 0.06	38.63^c^ ± 2.37	106.8 ± 12.02	74.10 ± 9.64
**8b**	**1**	5.02	6.35	1.33	40.10	114.10	84.80
	**2**	4.96	6.40	1.44	33.60	86.90	61.90
	**3**	5.02	6.45	1.43	32.90	93.30	58.80
**Mean ± SD**		5.0^abc^ ± 0.03	6.40^bc^ ± 0.05	1.40^ac^ ± 0.06	35.53^c^ ± 3.97	98.10^c^ ± 14.22	68.50 ± 14.20
**8c**	**1**	5.02	5.90	0.88	60.40	171.80	127.60
	**2**	4.97	5.80	0.83	61.80	159.50	113.60
	**3**	5.01	5.80	0.79	62.90	178.70	112.60
**Mean ± SD**		5.0^abc^ ± 0.03	5.83^ac^ ± 0.06	0.83^ab^ ± 0.05	61.70^b^ ± 1.25	170.0 ± 9.73	117.9 ± 8.39
**8d**	**1**	5.02	5.95	0.93	58.10	165.40	122.90
	**2**	4.96	6.03	1.07	50.70	131.0	93.20
	**3**	5.02	6.02	1.0	53.10	150.70	95.0
**Mean ± SD**		5.0^abc^ ± 0.03	6.0^abc^ ± 0.04	1.0^ab^ ± 0.07	53.97^b^ ± 3.78	149.0 ± 17.26	103.7 ± 16.65
**8e**	**1**	5.35	6.25	0.90	59.50	169.20	125.70
	**2**	5.25	6.35	1.10	49.30	127.40	90.70
	**3**	5.40	6.40	1.0	53.10	150.70	95.0
**Mean ± SD**		5.33^abc^ ± 0.08	6.33^bc^ ± 0.08	1.0^ab^ ± 0.10	53.97^b^ ± 5.15	149.1 ± 20.95	103.8 ± 19.09
**8f**	**1**	5.15	6.25	1.10	50.50	143.60	106.70
	**2**	5.15	6.35	1.20	44.70	115.50	82.20
	**3**	5.20	6.40	1.20	43.70	124.0	78.20
**Mean ± SD**		5.17^abc^ ± 0.03	6.33^bc^ ± 0.08	1.17^a^ ± 0.06	46.30 ± 3.67	127.7 ± 14.41	89.03 ± 15.43
**8g**	**1**	5.10	6.15	1.05	52.70	150.0	111.40
	**2**	5.15	6.10	0.95	56.20	145.20	103.40
	**3**	5.25	6.25	1.0	53.10	150.70	95.0
**Mean ± SD**		5.17^abc^ ± 0.08	6.17^abc^ ± 0.08	1.0^ab^ ± 0.05	54.0^b^ ± 1.92	148.6 ± 2.99	103.3 ± 8.20
**8h**	**1**	5.02	6.05	1.03	53.60	152.60	113.30
	**2**	4.96	6.15	1.19	45.20	116.70	83.10
	**3**	5.02	6.0	0.98	54.0	153.30	96.60
**Mean ± SD**		5.0^abc^ ± 0.03	6.07^abc^ ± 0.08	1.07^ab^ ± 0.11	50.93^b^ ± 4.97	140.9 ± 20.93	97.67 ± 15.13
**8i**	**1**	5.30	6.02	0.72	67.60	192.30	142.90
	**2**	5.30	5.93	0.63	71.0	183.30	130.50
	**3**	5.40	5.95	0.55	74.20	210.70	132.80
**Mean ± SD**		5.33^abc^ ± 0.06	5.97^abc^ ± 0.05	0.63^abc^ ± 0.09	70.93^bc^ ± 3.30	195.4^c^ ± 13.97	135.4 ± 6.60

^a^Statistically significant difference in comparison with control group.

^b^Statistically significant difference in comparison with celecoxib reference group.

^c^Statistically significant difference in comparison with diclofenac reference group.

##### *In vivo* gastric ulcerogenic activity

**Figure 6.**
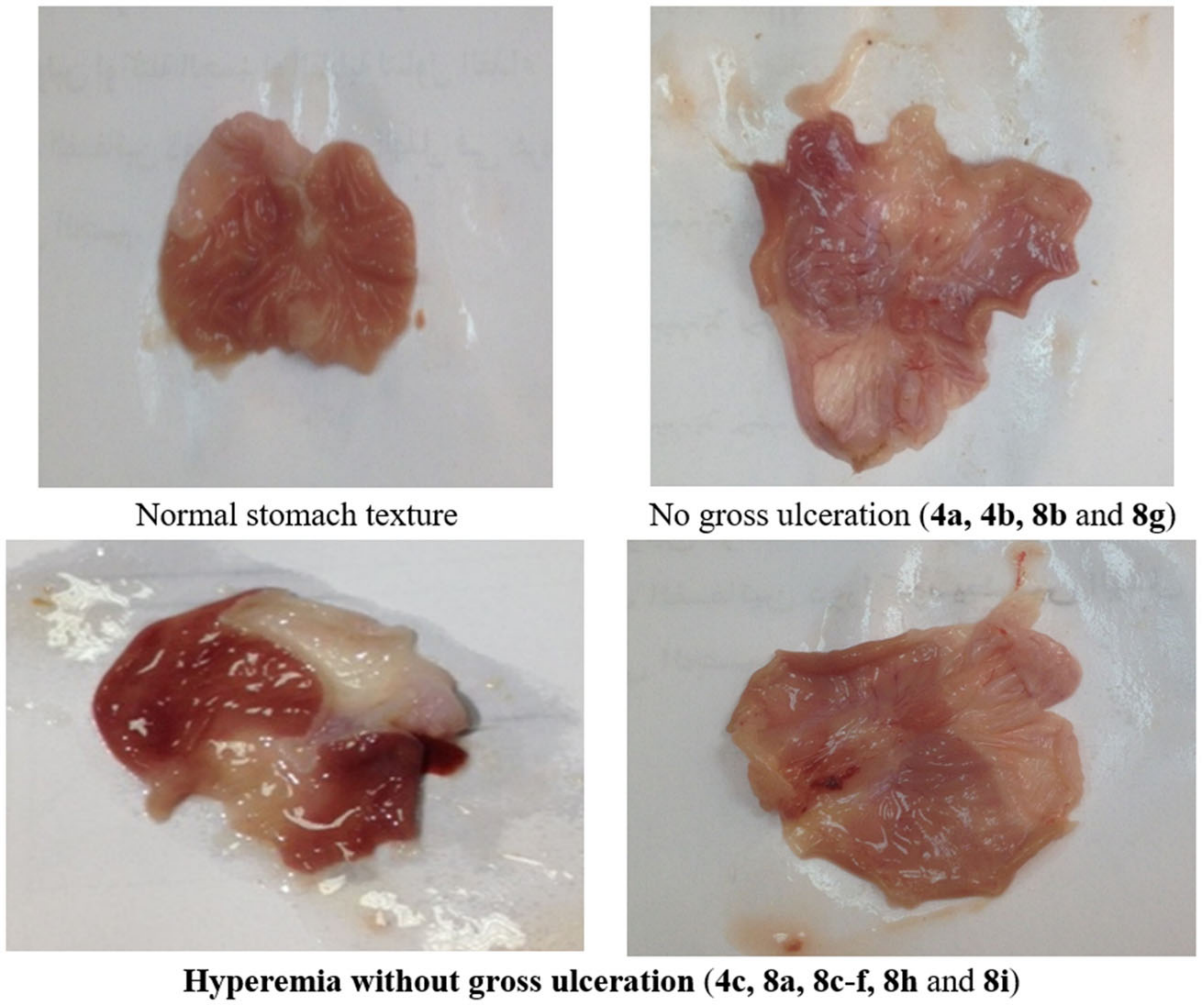
Gross appearance of gastric mucosa.

Compounds **4a–c** and **8a–i** were evaluated for their ulcerogenic potential in rats. Gross examination revealed that compounds **4a, 4b, 8b,** and **8g** showed superior gastrointestinal safety profile (no ulceration) as the references celecoxib and diclofenac sodium in the population of fasted rats. On the other hand, compounds **4c, 8a, 8c–f, 8h,** and **8i** showed variable degrees of hyperaemia (Figure 6).

### Structure–activity relationship

The *in vitro* inhibition of COX-1/2 and 5-LOX assays and the *in vivo* anti-inflammatory testing showed that, substitution at para position of phenyl at position 5 of pyrazole by either electron donating group (OCH_3_) or electron withdrawing group (Br) or unsubstituted did not affect the activity. This could be due to the presence of the phenyl ring out of coplanarity with the pyrazole moiety so could not affect the electronic configuration of the whole molecule hence binding affinity and activity. On the other hand, substitution at N1 of pyrazole affected the activity. The unsubstituted derivative **4a** showed *in vitro* COX-2/5-LOX inhibitory activity and *in vivo* potent anti-inflammatory activity. Furthermore, the activities of N1 aryl substituted derivatives were affected with the type of substitution at para position of N1 phenyl moiety. The substitution with either sulphonamide group or chloro showed higher *in vitro* COX-2/5-LOX inhibitory activity and *in vivo* anti-inflammatory activity than unsubstituted derivatives. This could be due to the importance of sulphonamide and chloro groups in polar and hydrophobic interactions with the active sites of the target COX-2/5-LOX enzymes.

### Molecular modelling

**Figure 7.**
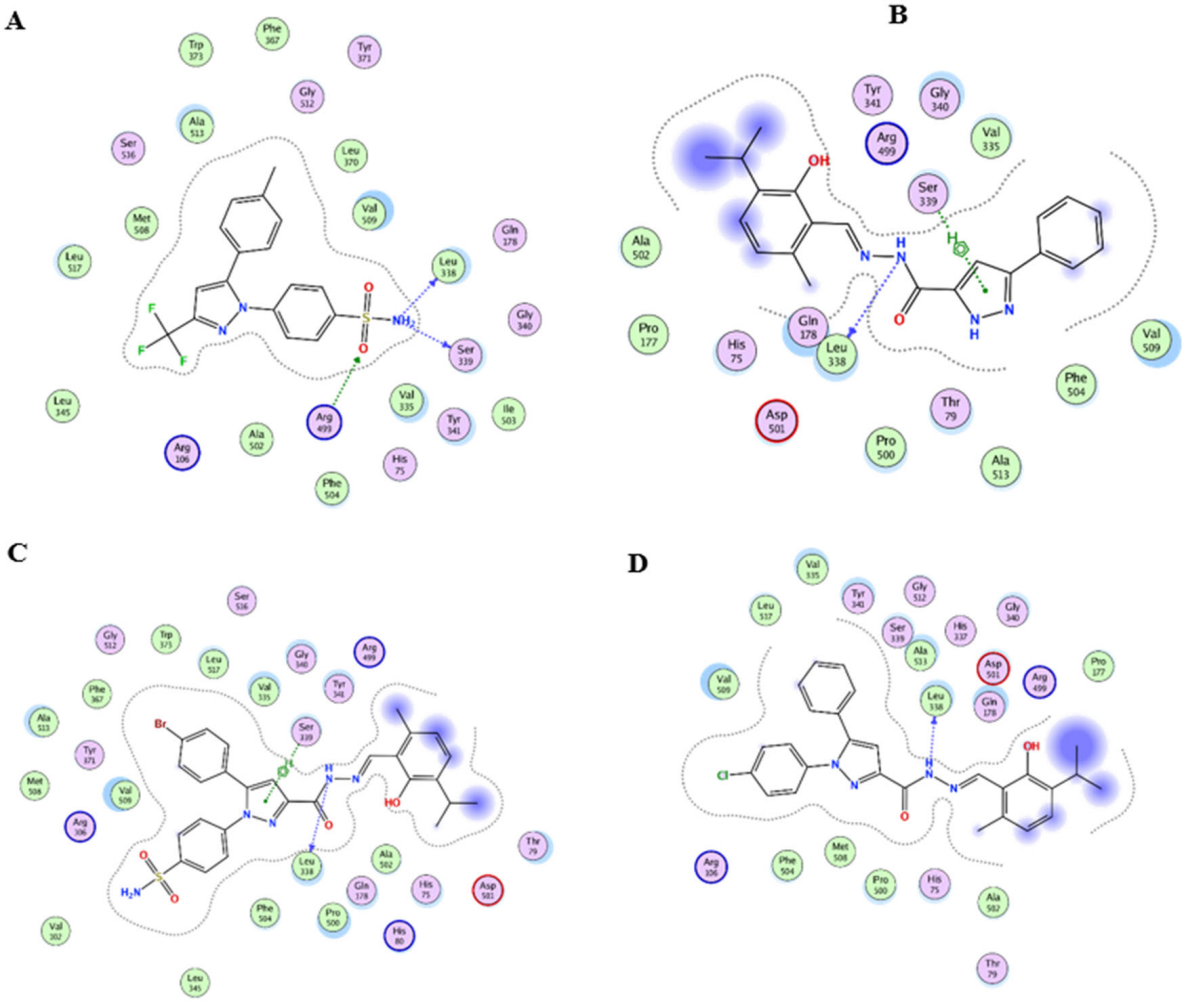
Mode of binding (2D) of celecoxib (A), **4a** (B), **8b** (C), and **8g** (D) inside the active site of COX-2.

**Figure 8.**
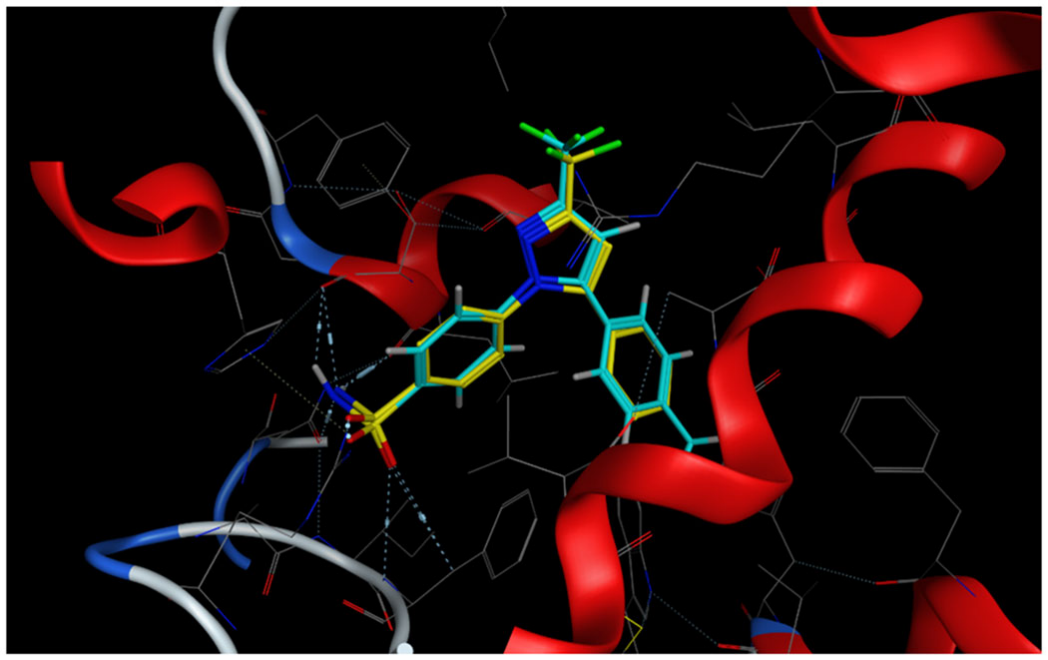
Overlay of celecoxib of crystallised (yellow) and docked (cyan) with RMSD = 0.422.

**Figure 9.**
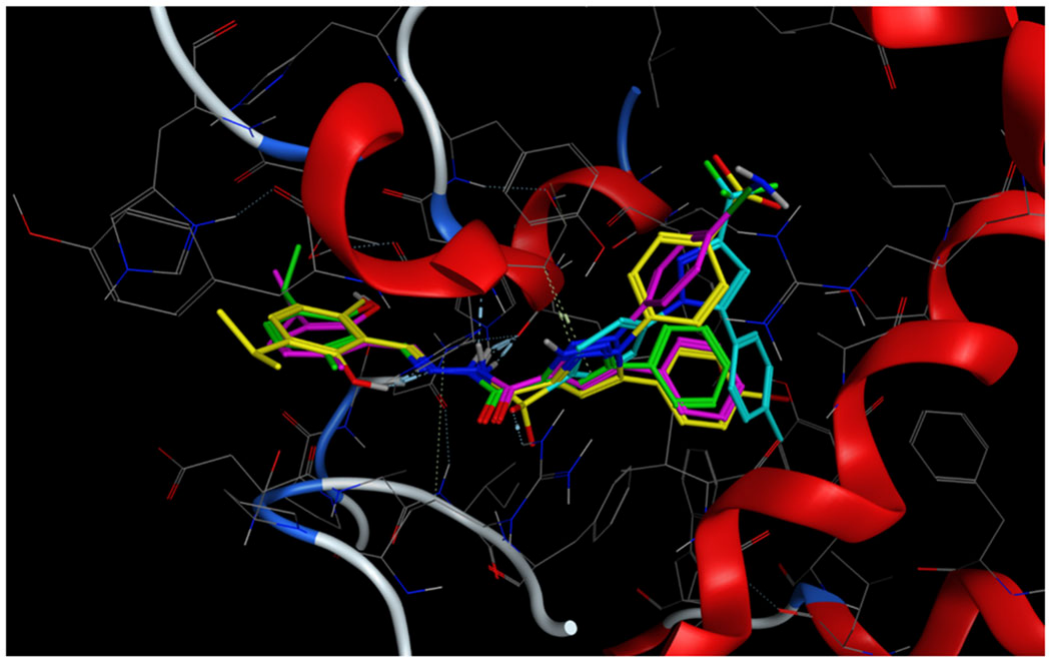
Overlay of compounds **4a** (green), **8b** (yellow), **8g** (pink), and celecoxib (cyan) inside the active site of COX-2.

**Figure 10.**
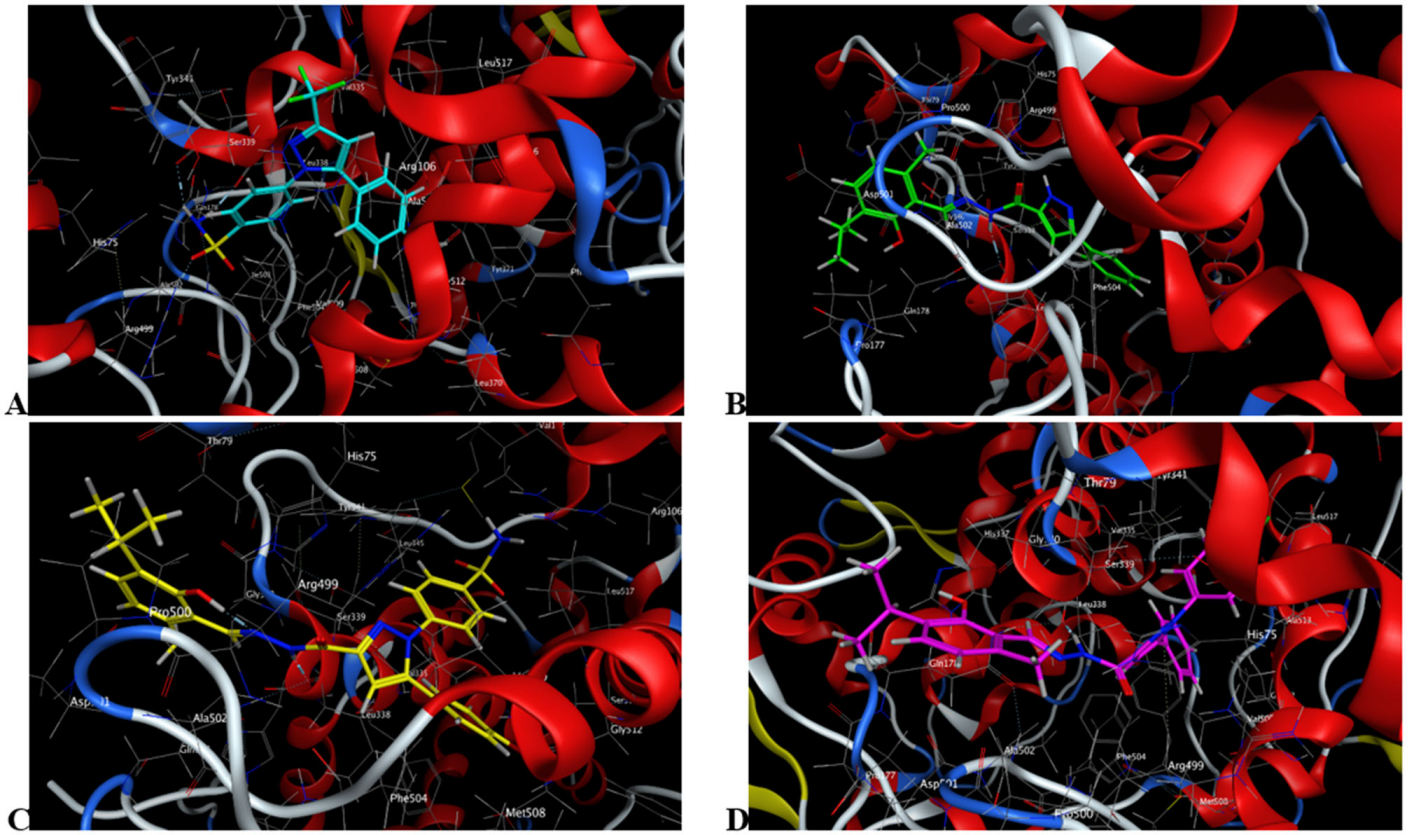
Mode of binding (3D) of celecoxib (A), **4a** (B), **8b** (C), and **8g** (D) into COX-2 active site.

**Figure 11.**
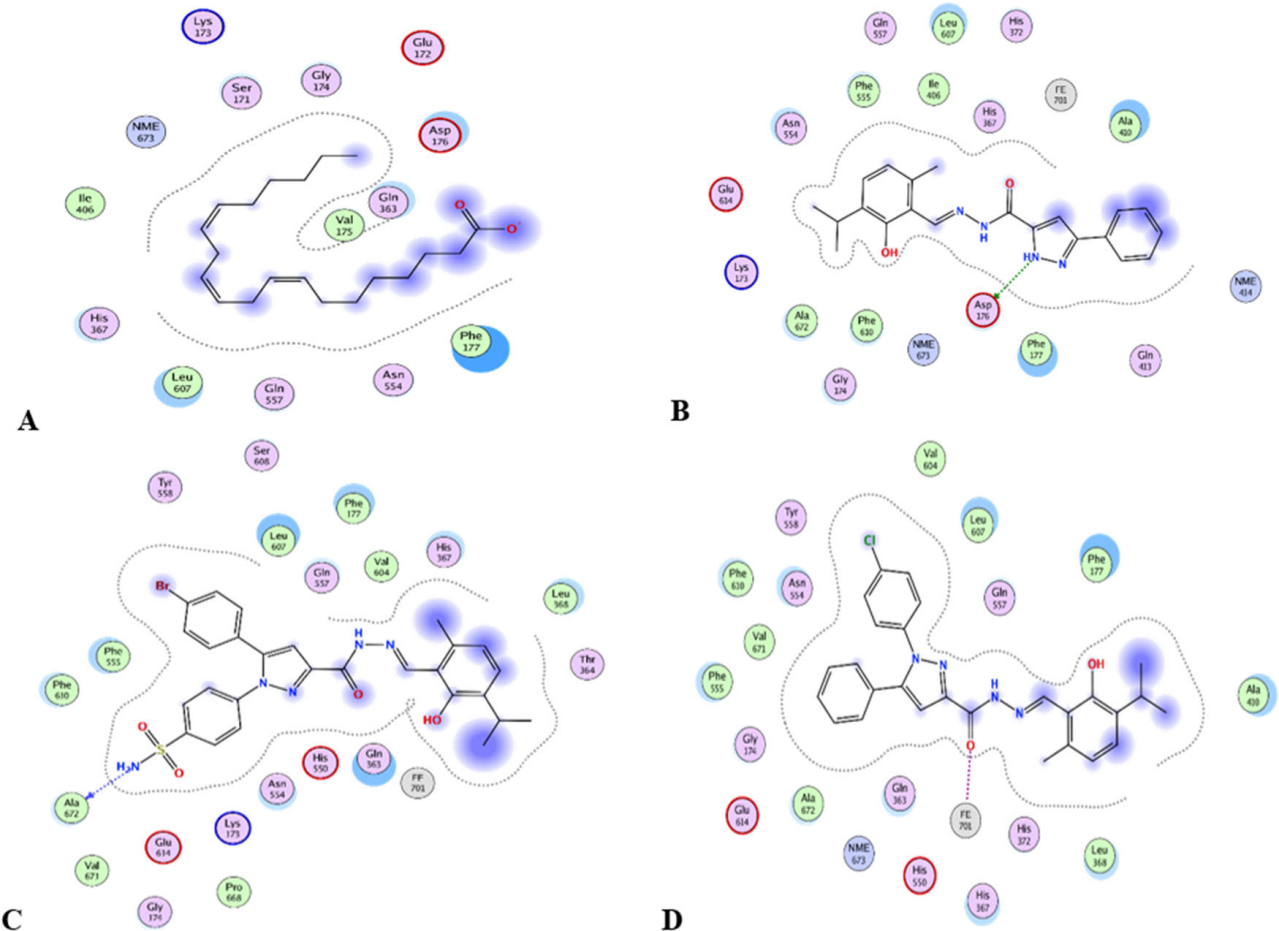
Mode of binding (2D) of Arachidonic acid (A), **4a** (B), **8b** (C), and **8g** (D) inside the active site of 5-LOX.

**Figure 12.**
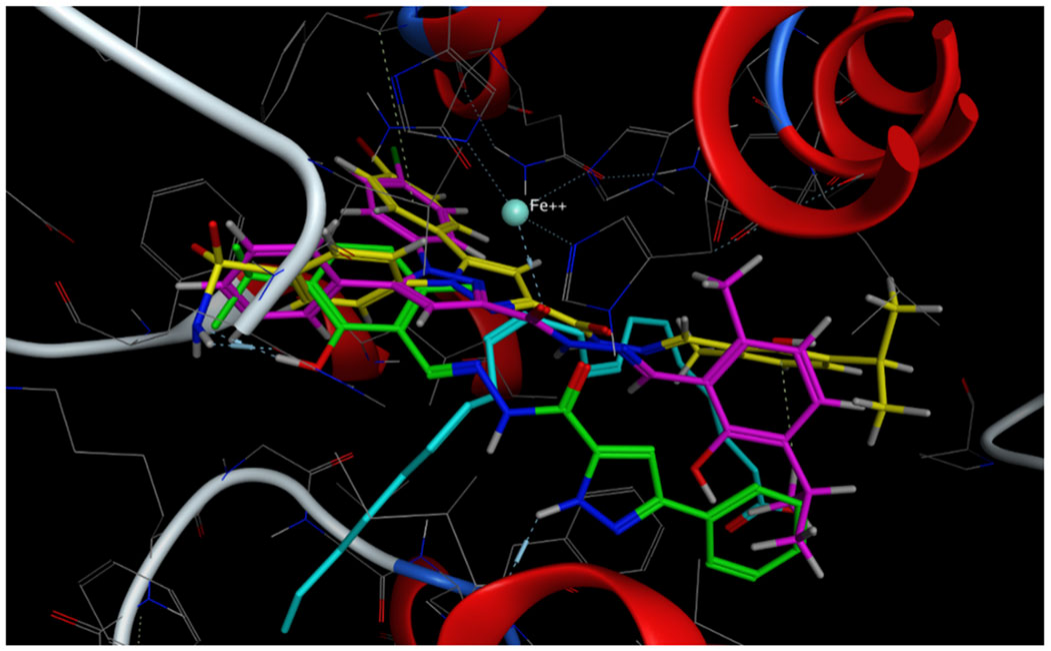
Overlay of compounds **4a** (green), **8b** (yellow), **8g** (pink), and arachidonic acid (cyan) inside the active site of 5-LOX.

**Figure 13.**
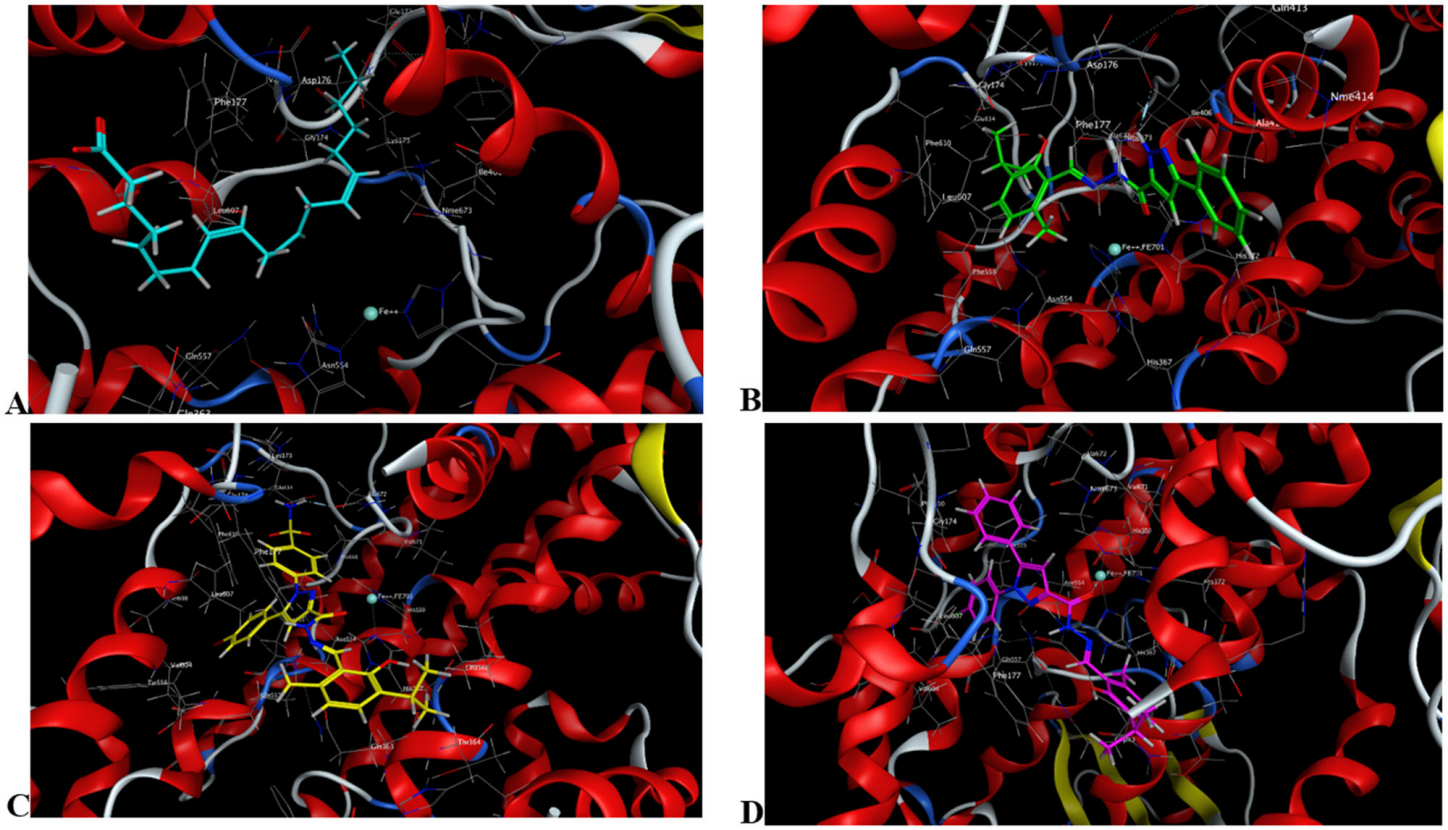
Mode of binding (3D) of arachidonic acid (A), **4a** (B), **8b** (C), and **8g** (D) inside the active site of 5-LOX.

MOE 2016.08 software^52^ was used for performing molecular modelling studies for the most active compounds **4a**, **8b,** and **8g**. These compounds were docked into the active site of COX-2 (PDB entry 3LN1) and 5-LOX (PDB entry 3V99). The results were illustrated in Tables 3 and 4 and Figures 7–13.

**Table 3.** Docking results of the active compounds in COX-2 active site.

Code	Score in Kcal/mol	Hydrogen bonds interaction amino acids	Hydrophobic interaction amino acids	Polar interaction amino acids
Celecoxib (Figures 7–10)	−9.32	Four bonds: two bonds between N of SO_2_NH_2_ and Ser339, Leu338; each O of SO_2_ with Arg499 and Ile503	Val335, Leu370, Phe367, Leu517, Leu345, Trp373, Val102, Met508, Ala502, Phe504, Val509	His75, Tyr341, Ser516, Tyr371, Gly340, Arg106, Gln178, Gly512, arene-H between phenyl of methylphenyl and Ala513
**4a** (Figures 7–9)	−7.79	One bond: between NH of CONH and *Leu338	Pro177, **Val335**, **Ala513, Ala513, Phe504, Ala502**, Pro500, **Val509**	Asp501, Thr79, **His75, Arg499, Gln178**, **Gly340, Tyr341**, arene-H between pyrazole and **Ser339**
**8b** (Figures 7–9)	−10.40	One bond: between NH of CONH and **Leu338**	**Val335, Leu517, Trp373, Met508, Val509, Ala513, Phe367, Phe504, Ala502,** Pro500, **Leu345, Val102**	**Gly512**, Asp501, Thr79, His80, **Arg499, Tyr341, His75, Gln178, Gly340, Ser516, Arg106, Tyr371**, arene-H between pyrazole and **Ser339**
**8g** (Figures 7–9)	−8.76	One bond: between NH of CONH and **Leu338**	Pro177, **Val335, Leu517, Met508, Ala513, Phe504, Ala502**, Pro500, **Val509**	**Gly512**, Asp501, Thr79, His337, **Arg499, Tyr341, His75, Gln178, Gly340, Arg106, Ser339**

*Amino acids interacted with the co-crystallised ligand (celecoxib) were marked in bold format.

**Table 4.** Docking results of the active compounds in 5-LOX active site.

Code	Score in Kcal/mol	Hydrogen bonds interaction amino acids	Hydrophobic interaction amino acids	Polar interaction amino acids
Co-crystallised ligand (arachidonic acid)	−9.42	No hydrogen bonds	Ile406, Phe177, Val175, Leu607.	Gln557, Gly174, Asn554, His367, Asp176, Gln363, Lys173, Ser171, Glu172
**4a** (Figures 10–12)	−7.19	One hydrogen bond between N1 of pyrazole and *Asp176	Ala410, Phe555, Ala672, **Phe177**, Phe610, **Leu607**, **Ile406**	**Asn554**, His372, **Gly174**, **His367**, Gln557, FE701, Glu614, **Lys173**, Gln413
**8b** (Figures 10–12)	−8.38	One hydrogen bond between N of sulphonamide and Ala672	Val604, Leu368, **Phe177**, Val671, Ala672, Phe610, **Leu607**, Phe555, Pro668.	Tyr558, Gln557, Ser608, **His367**, His550, **Gln363**, Glu614, **Gly174, Asn554**, Lys173, FE701, Thr364.
**8g** (Figures 10–12)	−7.78	Coordinate bond between carbonyl oxygen and FE701.	**Leu607**, Val604, Phe555, Phe610, Val671, **Phe177**, Ala672, Ala410, Leu368.	Glu614, **Gly174, Gln363**, Tyr558, **His367**, Gln557, **Asn554**, His550, His372.

*Amino acids interacted with the co-crystallised ligand (Arachidonic acid) were marked in bold format.

#### Docking to COX-2 active site

Compounds **4a**, **8b,** and **8g** which showed dual *in vitro* COX-2/5-LOX inhibiting activity and the reference celecoxib were docked into COX-2 active site (pdb entry 3LN1)^56^ using MOE version 2016.0802 software^52^. The docking solutions of the compounds **4a**, **8b, 8g,** and celecoxib (Table 3 and Figures 7–10) confirmed the potential activities against COX-2 as the mode of interactions of the best poses were comparable to the reference celecoxib. All the target compounds interacted with hydrogen bond with the key amino acid Leu338. In addition, they showed potential polar interaction with the key amino acids Arg499 and Ser339^57^ in the polar side pocket of COX-2 active site. Interaction with this small pocket’s amino acids is essential for the selective inhibition of the enzyme^57^. Moreover, overlay of compounds **4a**, **8b, 8g,** and celecoxib inside the active site of COX-2 (Figure 9) revealed that, the pyrazole moiety of **4a** and 1-arylpyrazole moiety of **8b** and **8g** were superposed on 1-arylpyrazole moiety of celecoxib with p-substituents interacted in the same position of trifluromethyl of celecoxib. Besides, the carbonyl group interacted in the same position of sulphonamide group of celecoxib.

#### Docking to 5-LOX active site

Compounds **4a**, **8b,** and **8g** which showed dual *in vitro* COX-2/5-LOX inhibiting activity and the co-crystallised ligand arachidonic acid were docked into 5-LOX active site (pdb entry 3V99)^58^ using MOE version 2016.0802 software^52^. The docking solutions of the compounds **4a**, **8b, 8g,** and arachidonic acid (Table 4 and Figures 11–13) supported the potential activities against 5-LOX . It was reported that, 5-LOX active site comprises several anchors. Polar positively-charged anchor consists of His550, His367 and His372 which interact with Fe^2+^. Polar anchor form both electrostatic and hydrogen-bonding interactions comprises Asp176, Asn180 and Gln363. Hydrophobic anchor contains Phe177, Leu607, Ile673, Leu414, Phe421, Gly174, Val175, Leu368, and Ile406^59^. Compounds **4a**, **8b**, and **8g** interacted with various anchors in 5-LOX active site. All of them showed polar interaction with His367 beside additional interaction with His372 for **4a** and His550 for **8b** and **8g**. Besides, all compounds elicited polar interaction with Gln363 in addition to hydrogen bond formed between N1-pyrazole of **4a** and Asp176 as part of the second polar anchor. Furthermore, **4a**, **8b,** and **8g** interacted by hydrophobic anchor with Phe177 and Leu607 in addition to hydrophobic interaction with Leu368 for **8b** and **8g** and Ile406 for **4a**. It is worth mentioning that, arachidonic acid did not form hydrogen bond with 5-LOX active site, while the target compounds formed hydrogen bond and coordinate bond interactions with 5-LOX active site so the target compounds could have favourable binding affinity towards 5-LOX more than arachidonic acid.

## Conclusion

Selective COX-2 inhibitors have many benefits in treatment of inflammation, but this selective inhibition resulted in accumulation of arachidonic acid at 5-LOX site which leads to overproduction of LTs which in turn induced asthmatic problems, gastric damage, and ulceration. New thymol − 1,5-disubstitutedpyrazole hybrids were synthesised as dual COX-2/5-LOX inhibitors to get safer anti-inflammatory therapy. Compounds **8b**, **8g**, **8c,** and **4a** displayed *in vitro* inhibitory activity against COX-2 nearly equal to celecoxib with high SI comparable to celecoxib. All compounds, **4a–c** and **8a–i**, showed 5-LOX inhibitory activity higher than reference quercetin. Furthermore, all compounds, **4a–c** and **8a–i**, showed *in vivo* inhibition of formalin induced paw oedema higher than celecoxib. Compound **4a** was the most potent with inhibition percentage higher than celecoxib and diclofenac sodium. In addition, compounds **4a, 4b, 8b,** and **8g** showed superior gastrointestinal safety profile (no ulceration) as the references celecoxib and diclofenac sodium in the population of fasted rats. *In silico* docking studies in the COX-2 and 5-LOX active sites predicted that, the target compounds could have strong binding affinity to the target enzymes active sites in comparison with the reference celecoxib and arachidonic acid, respectively. In conclusion, compounds **4a**, **8b,** and **8g** achieved the target goal. They elicited *in vitro* dual inhibition of COX-2/5-LOX higher than celecoxib and quercetin, *in vivo* potent anti-inflammatory activity higher than celecoxib and *in vivo* superior gastrointestinal safety profile (no ulceration) as celecoxib.

## Supplementary Material

Supplemental Material
