# Supplementary material for: Towards safer anti-inflammatory therapy: synthesis of new thymol–pyrazole hybrids as dual COX-2/5-LOX inhibitors
Source: J Enzyme Inhib Med Chem. 2022 Nov 21;38(1):294–308. doi: 10.1080/14756366.2022.2147164 (PMC9704104; doi:10.1080/14756366.2022.2147164)
Supplement: Supplemental Material [file IENZ_A_2147164_SM4879.pdf]

Towards Safer Anti-inflammatory Therapy: Synthesis of New Thymol – 1,5-Diarylpyrazole Hybrids as Dual COX-2/5-LOX Inhibitors

Chemistry

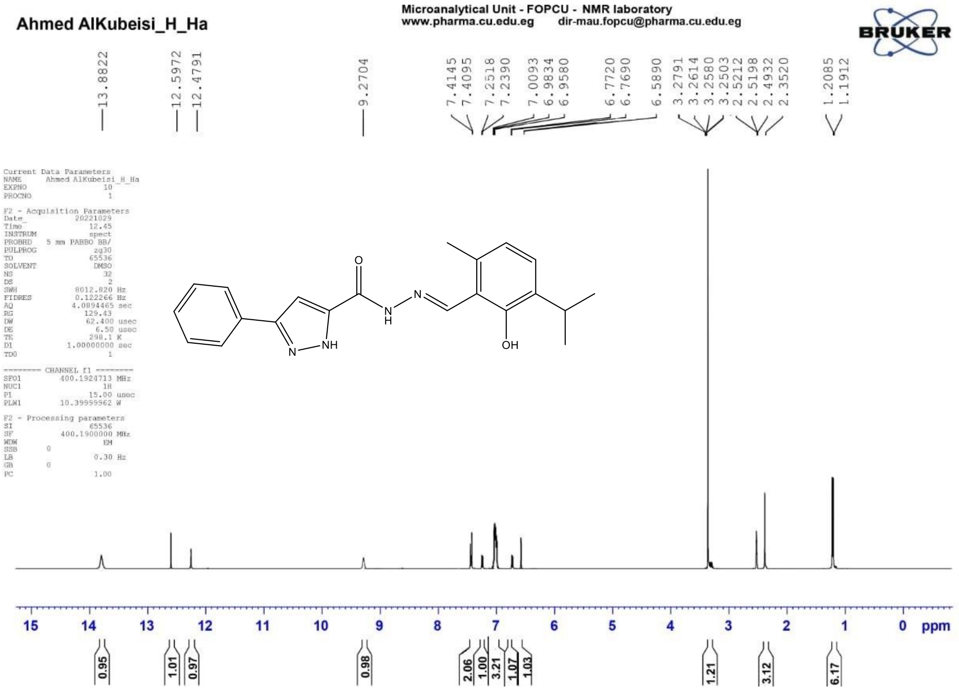

^{1}H-NMR of Compound 4a

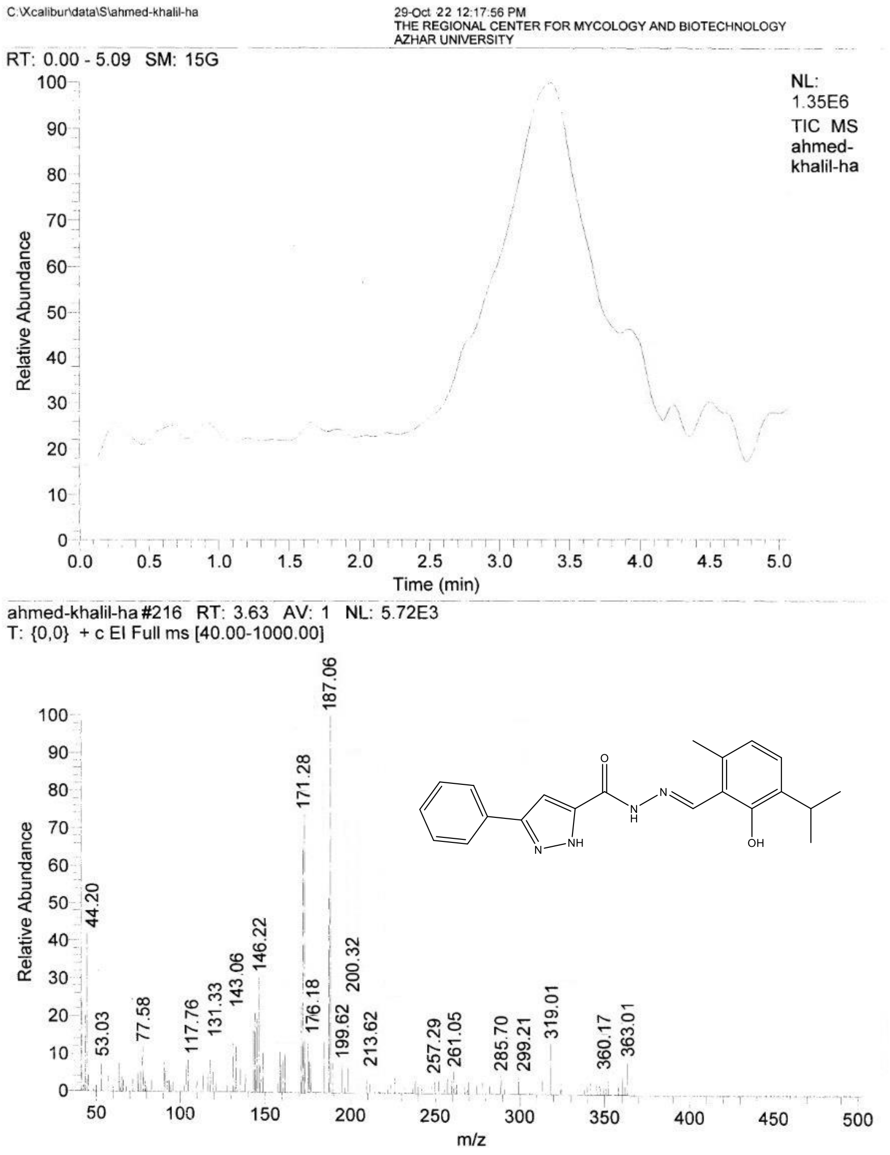

Mass spectroscopy of Compound 4a

Al-Azhar University

The Regional Center for Mycology and Biotechnology

Requester Data:

Name: Dr. Ahmed Khalil Ibrahim
Authority: Faculty of Pharmacy,
Alexandria University

Sample Data:

Four samples had been submitted for elemental analysis.

Analysis Report:

Sample Code	C%	H%	N%	S%
Ha	69.26	5.88	15.09	-
HC	66.98	6.45	14.57	-
Gb_1	51.98	3.34	7.28	6.76
Gb_2	49.38	3.23	14.50	5.42

INVESTIGATOR

DIRECTOR

Al-Azhar University Campus - Nasr City, Cairo, Egypt.
Tel: 0202 22620373 Fax: 0202 22620373
E.mail: rcmb@azhar.edu.eg
Website: http://www.azhar.edu.eg.htm * http://www.azhar.edu.eg/pages/fungi_center.htm
Facebook: RCMB AZHAR P.O. box mail : 11751 Nasr City Cairo, Egypt.

Elemental analysis

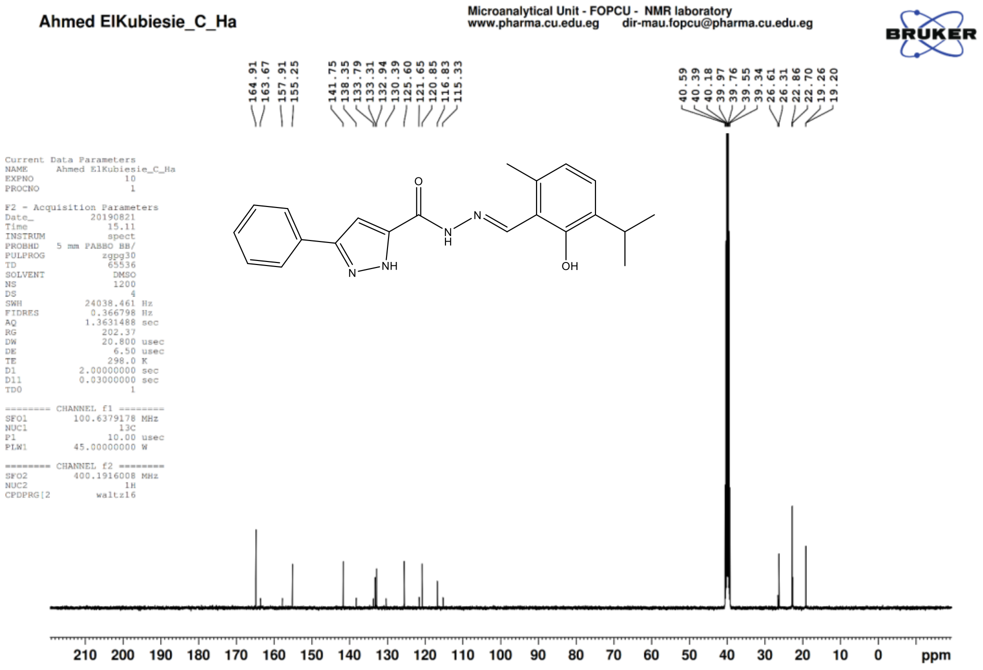

13C-NMR of Compound 4a

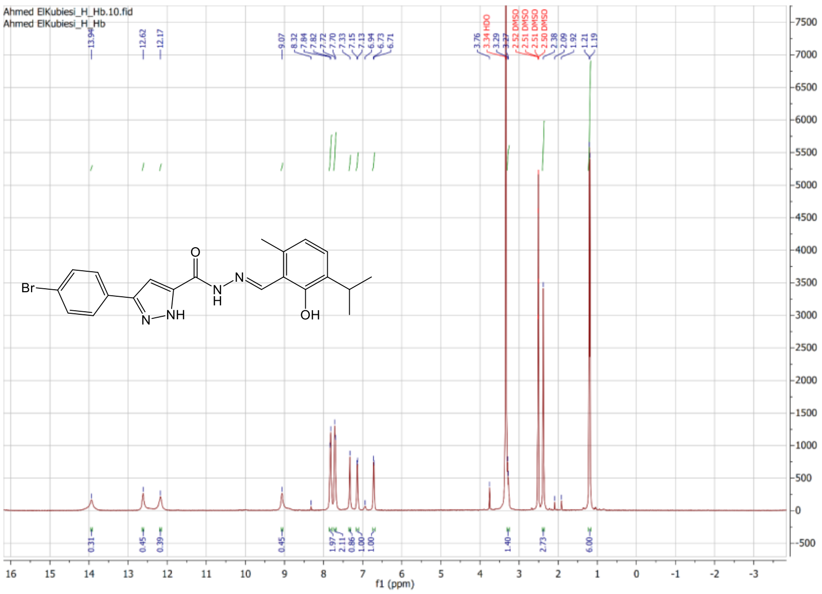
Ahmed ElKubiesi_H_Hb.10.fid
Ahmed ElKubiesi_H_Hb
Br
O
N
H
N
NH
OH
f1 (ppm)

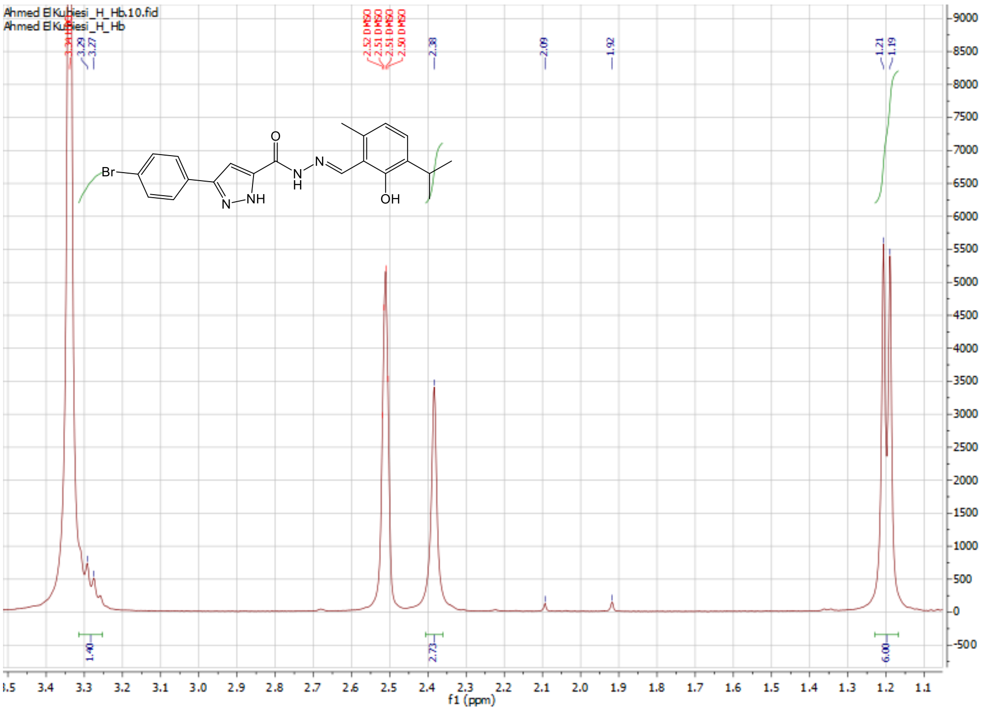
Ahmed ElKubiesi_H_Hb.10.fid
Ahmed ElKubiesi_H_Hb
Br
O
N
H
N
NH
OH
f1 (ppm)

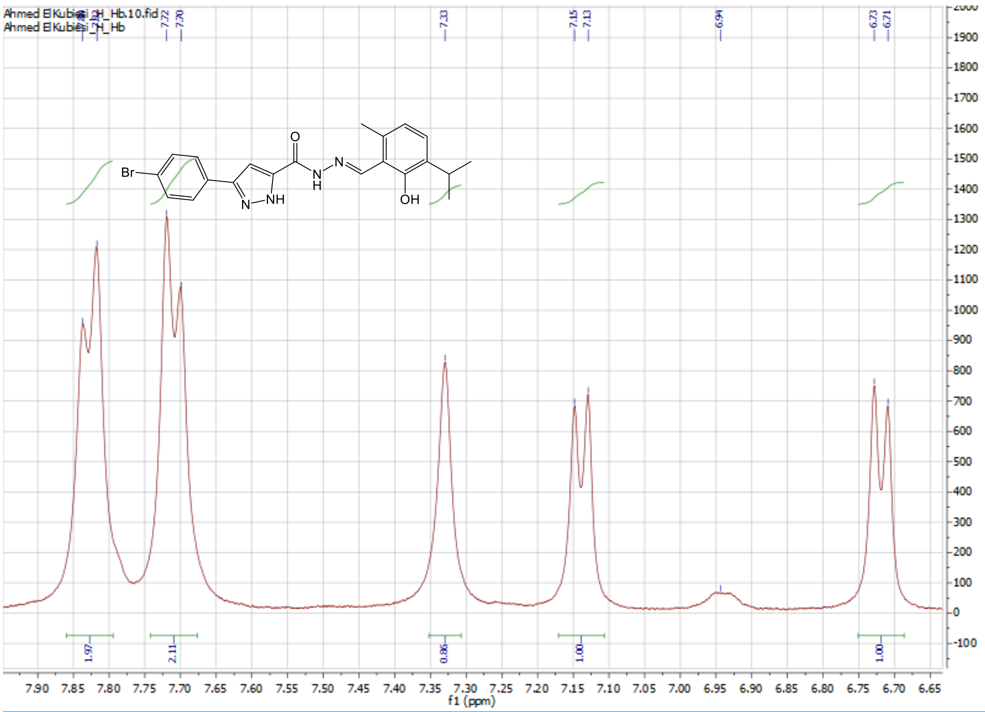

H-NMR of Compound 4b

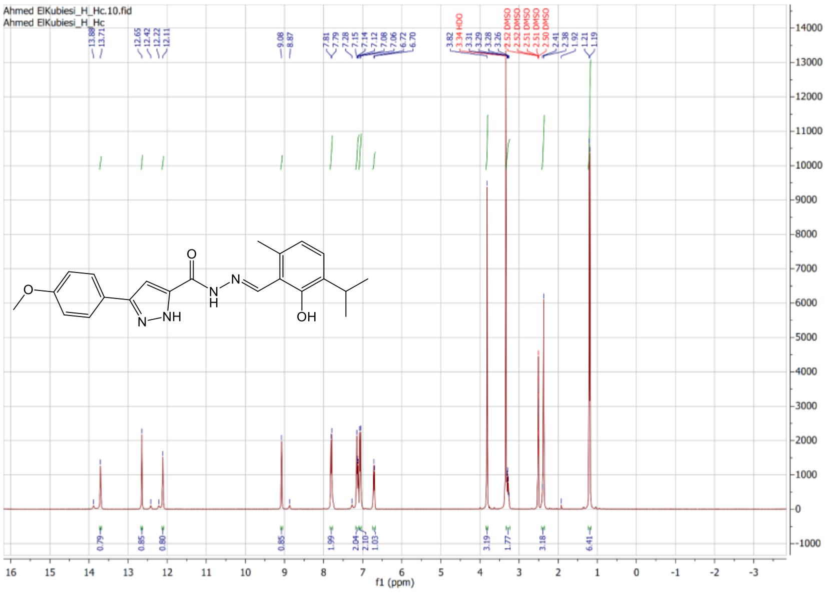

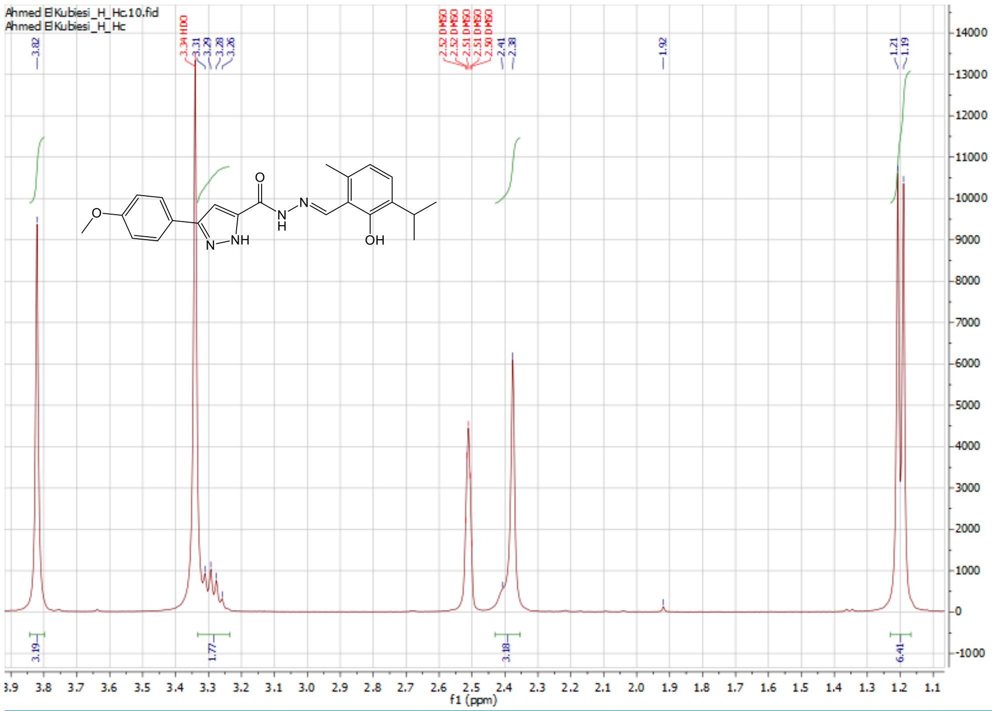

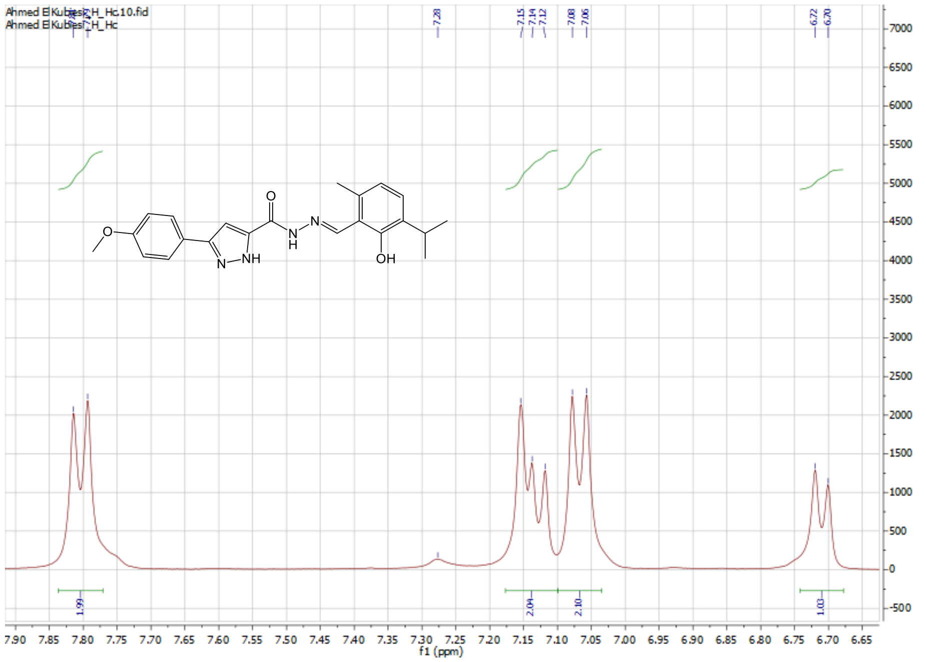

H-NMR of Compound 4c

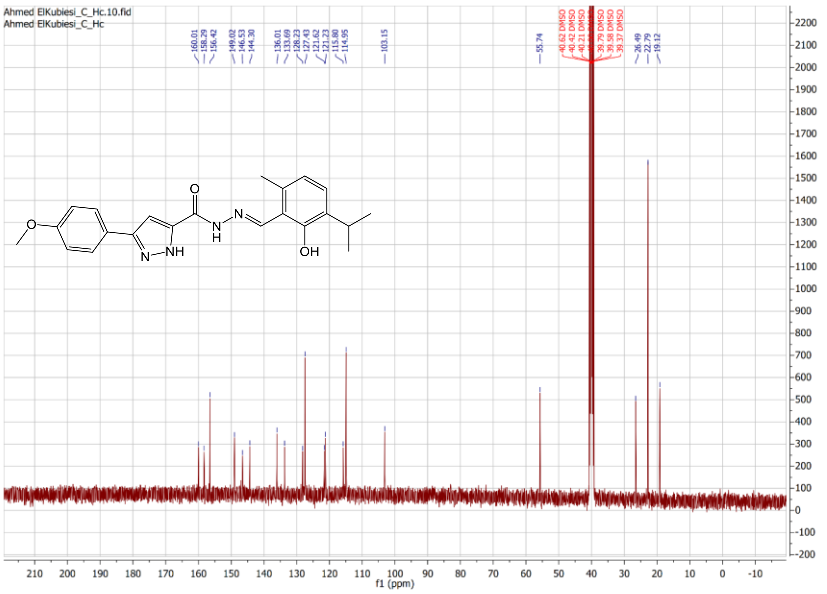

13C-NMR of Compound 4c

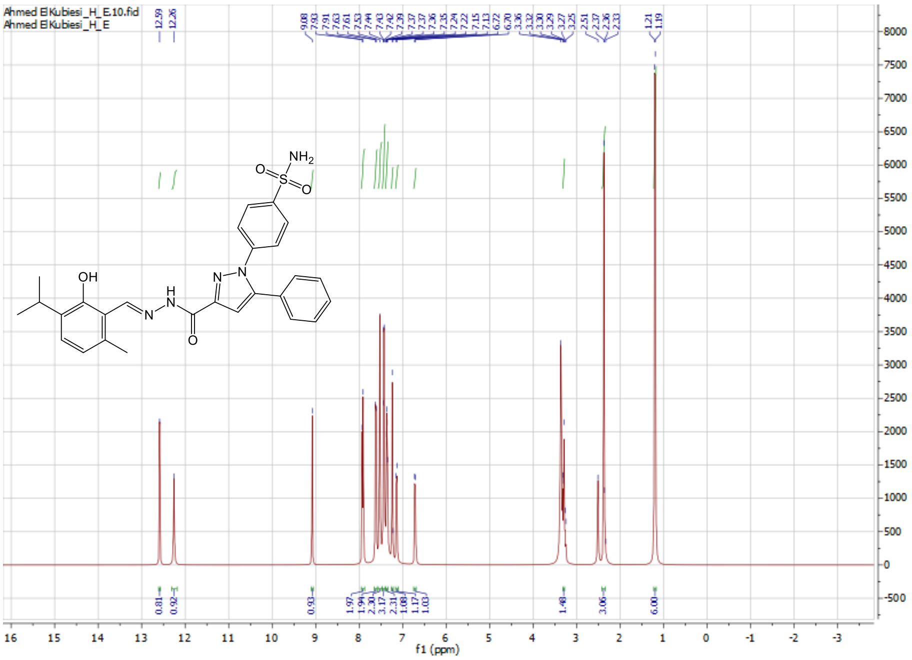

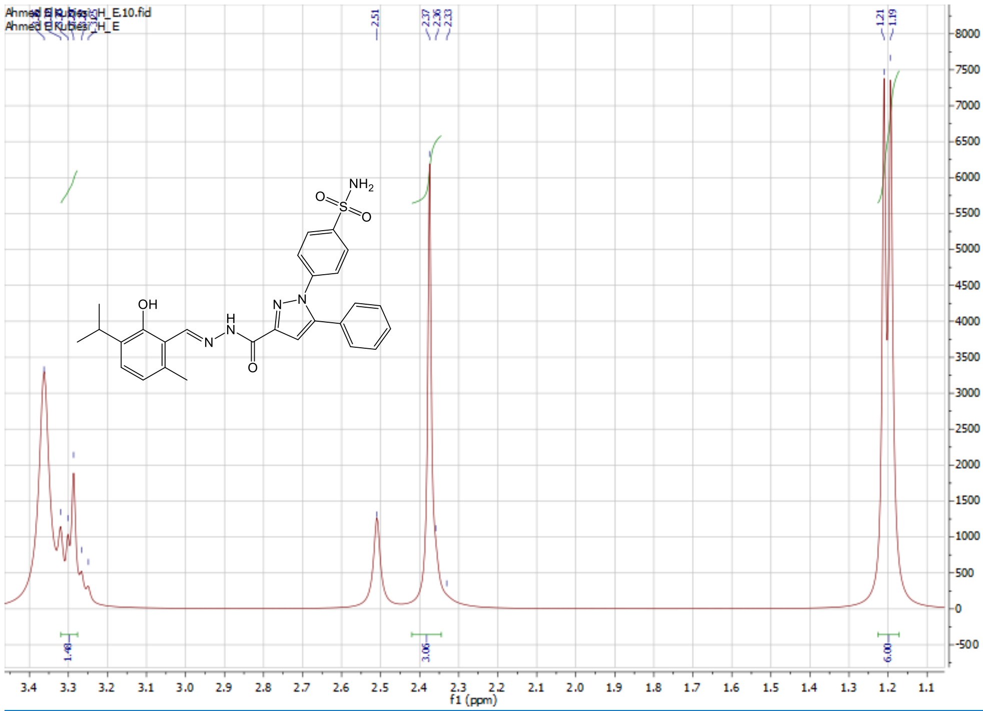

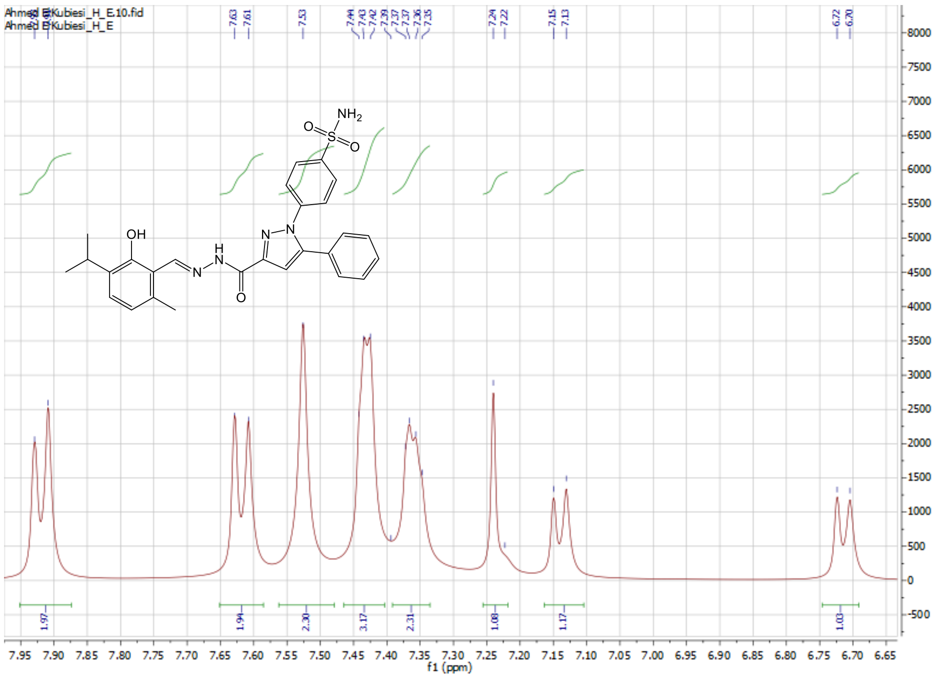

H-NMR of Compound 8a

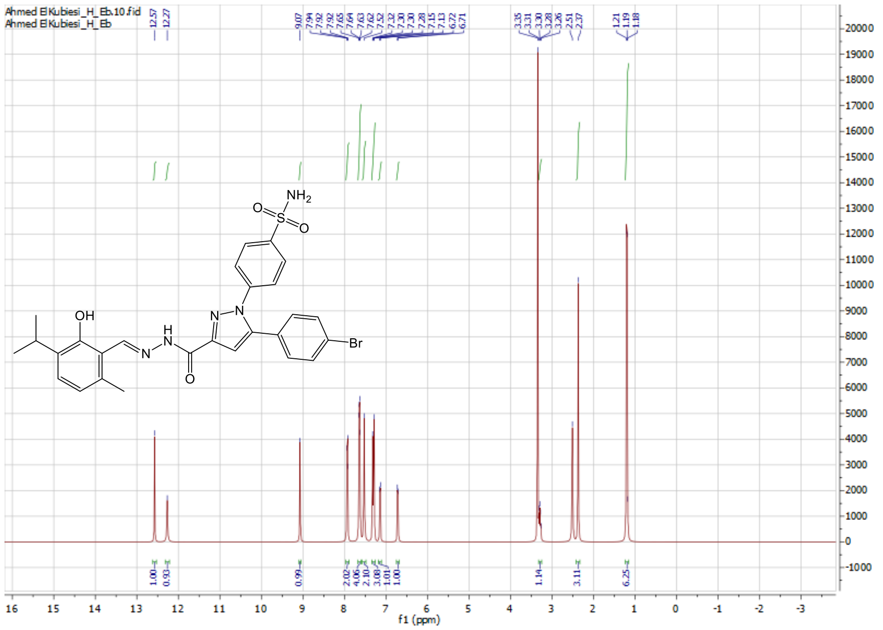

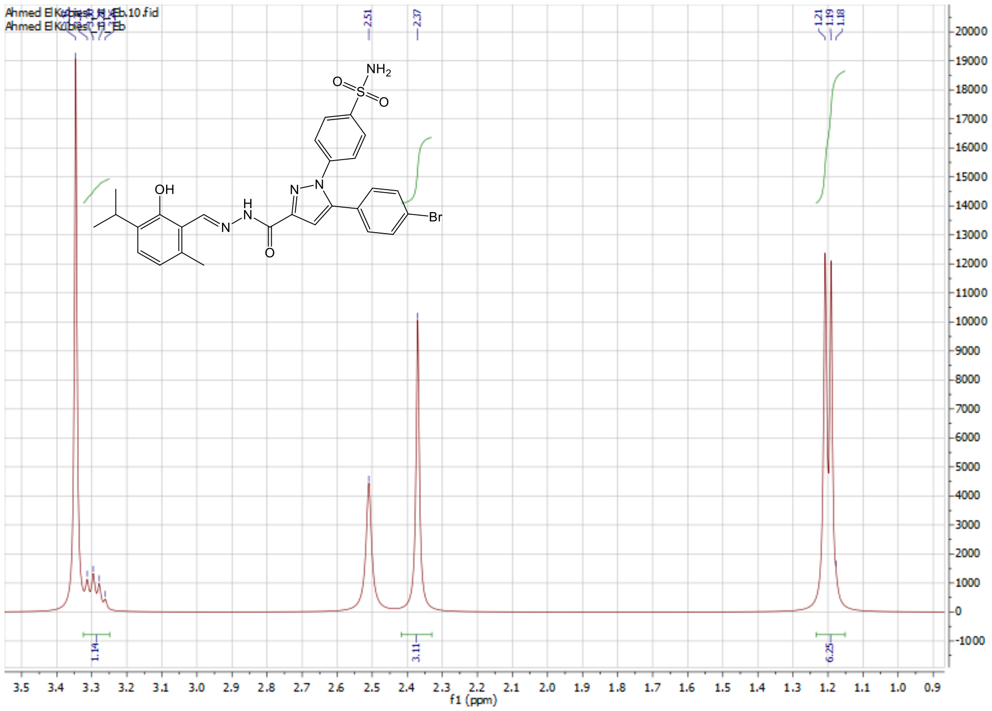

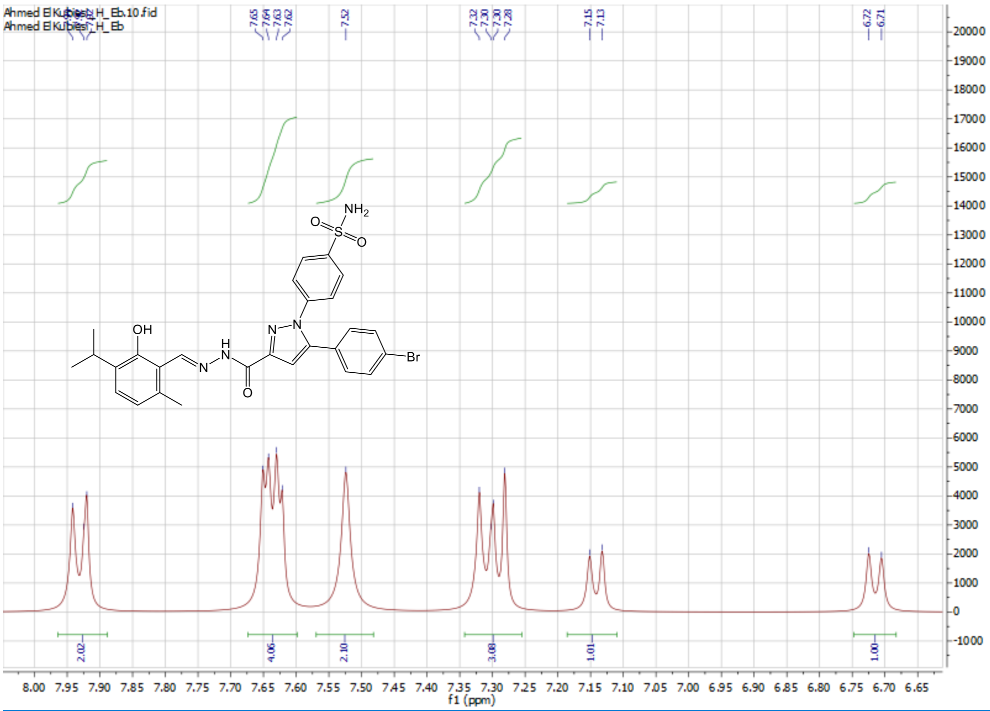

H-NMR of Compound 8b

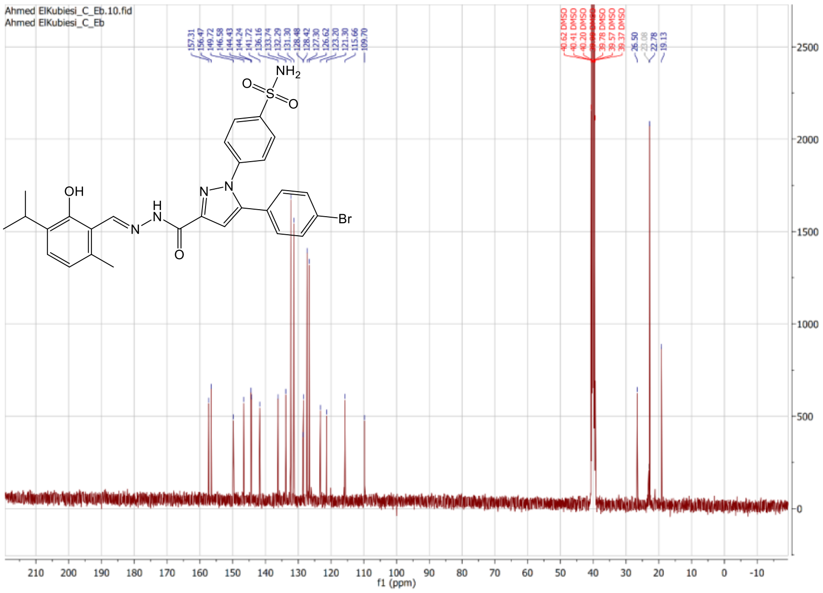

13C-NMR of Compound 8b

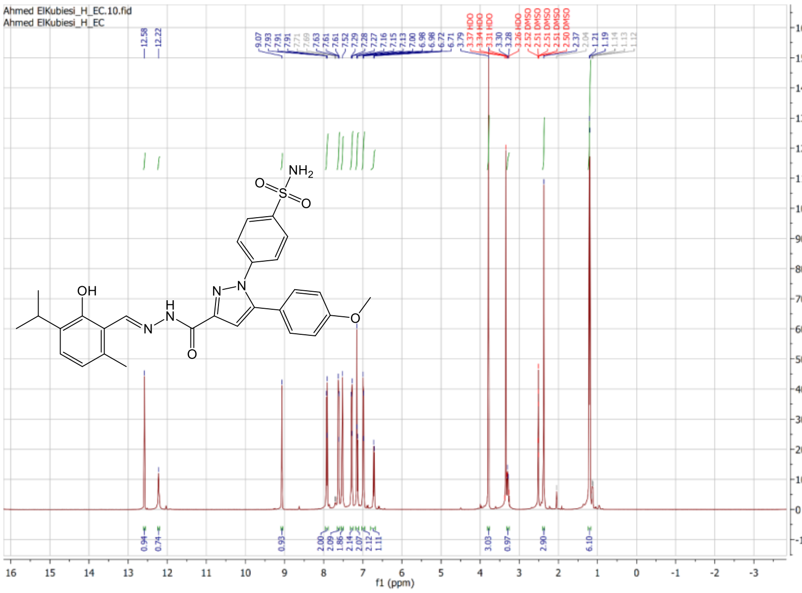
Ahmed ElKubiesi_H_EC.10.fid
Ahmed ElKubiesi_H_EC
f1 (ppm)

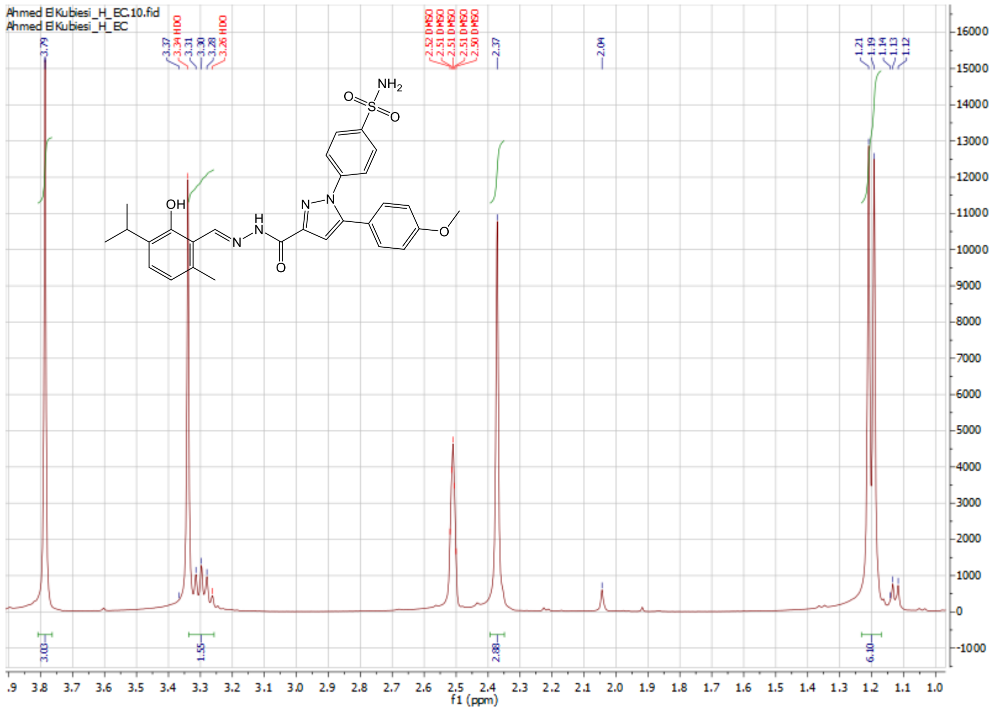
Ahmed El Kubiesi_H_EC.10.fid
Ahmed El Kubiesi_H_EC
f1 (ppm)

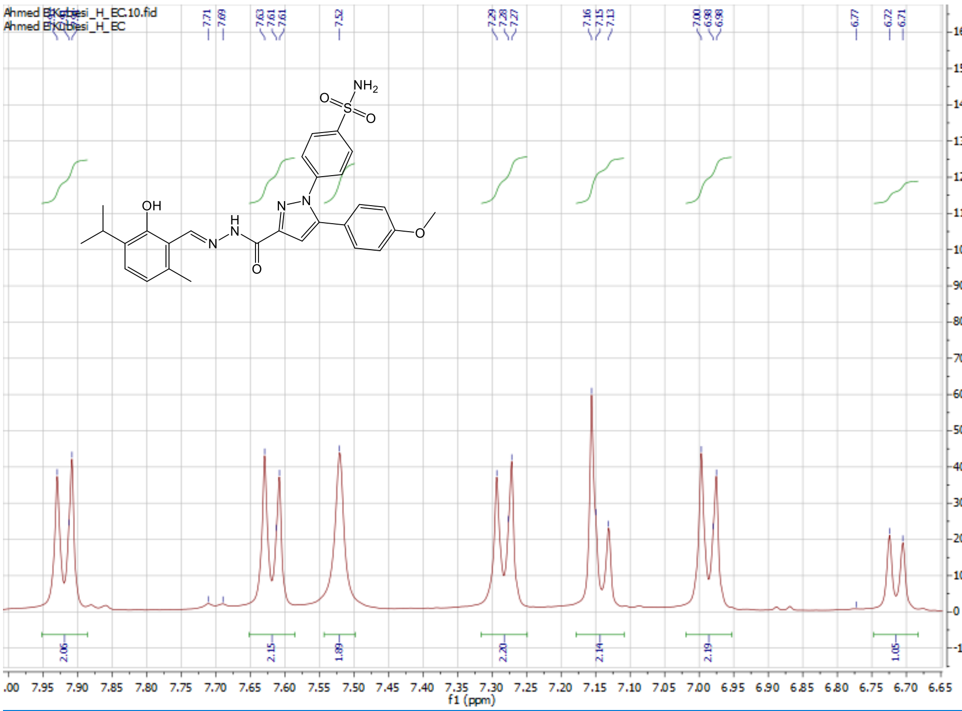

H-NMR of Compound 8c

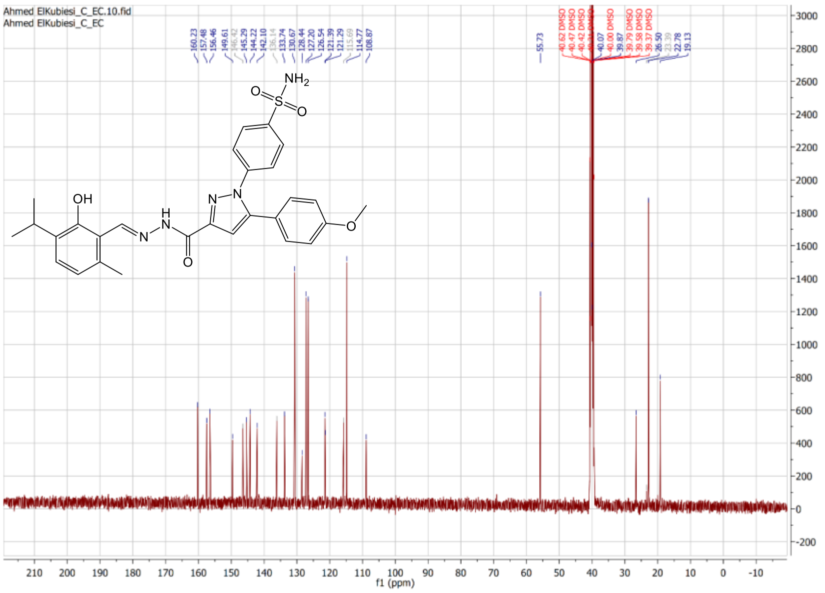

13C-NMR of Compound 8c

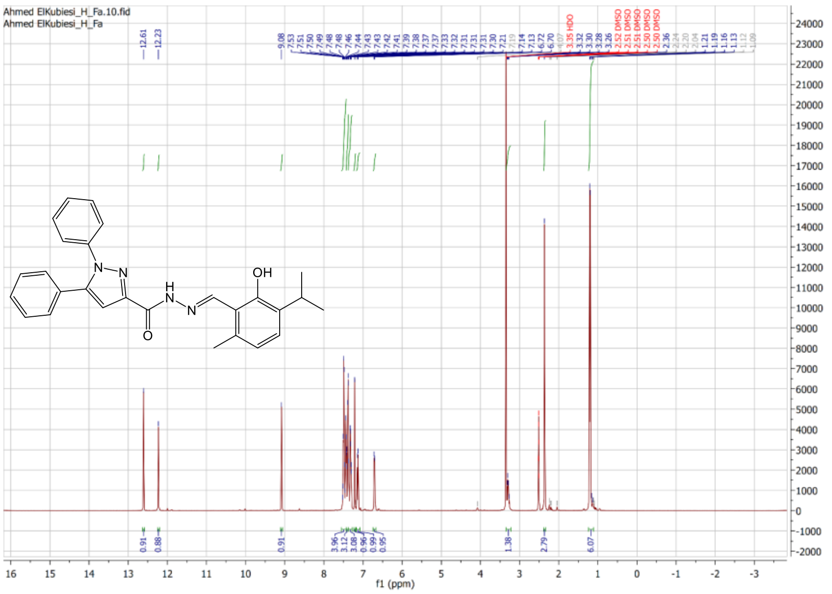

Ahmed ElKubiesi_H_Fa.10.fid
Ahmed ElKubiesi_H_Fa
OH
f1 (ppm)

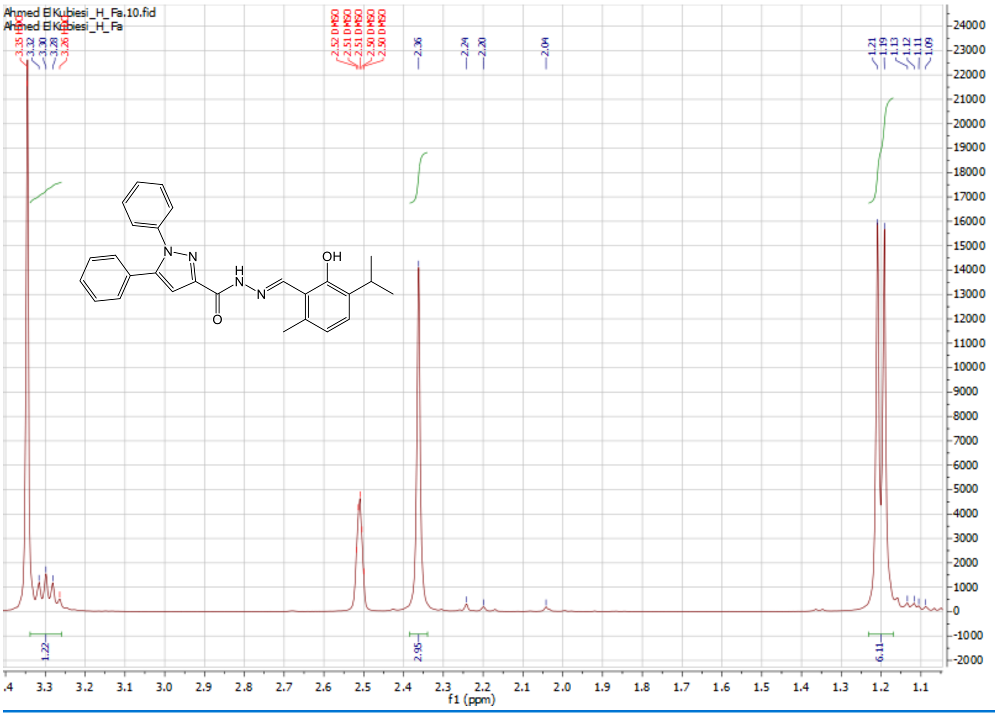

Ahmed ElKubiesi_H_Fa.10.fid
Ahmed ElKubiesi_H_Fa
OH
f1 (ppm)

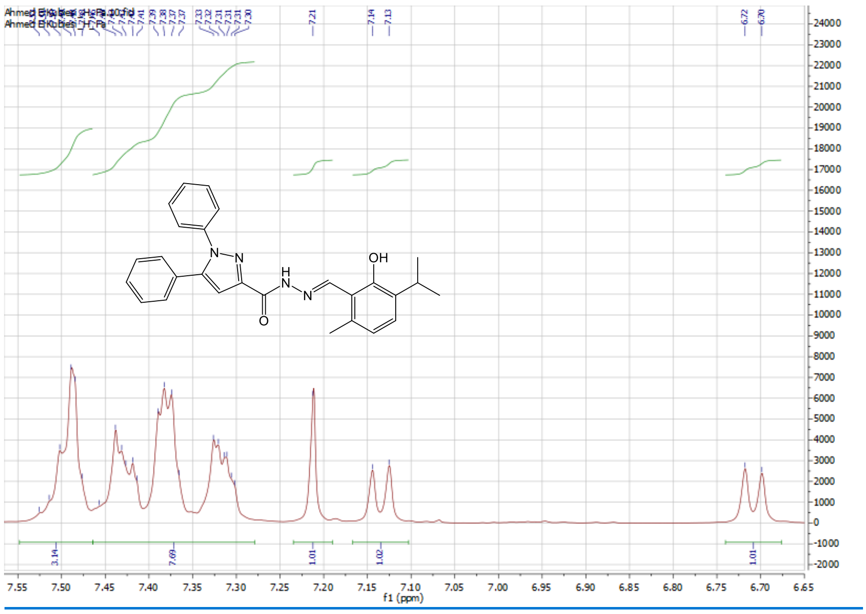

H-NMR of Compound 8d

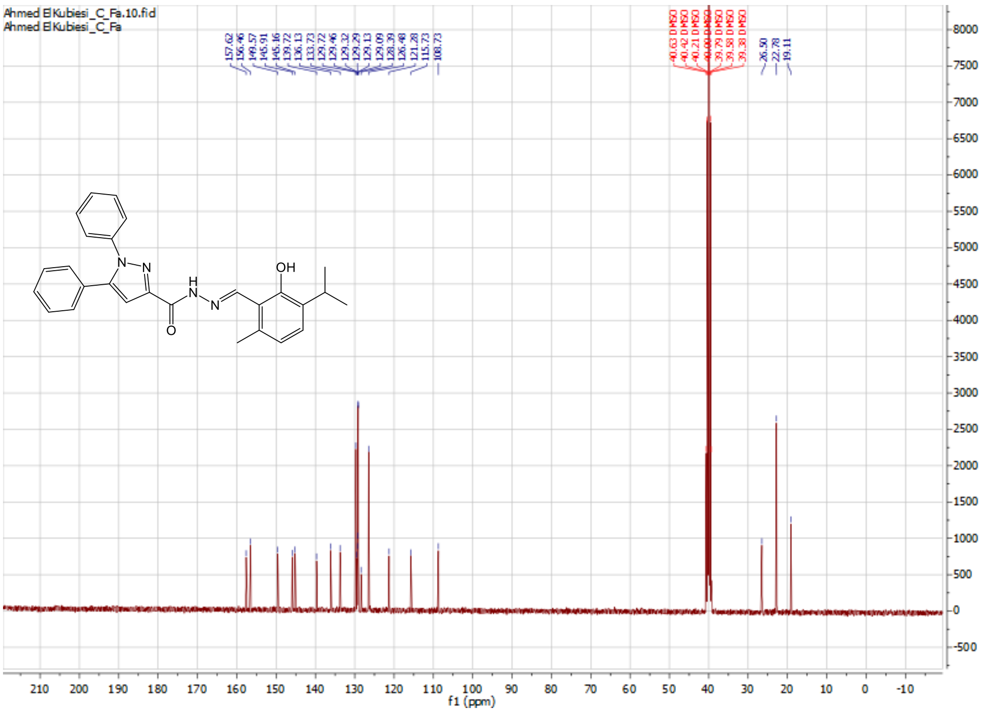

13C-NMR of Compound 8d

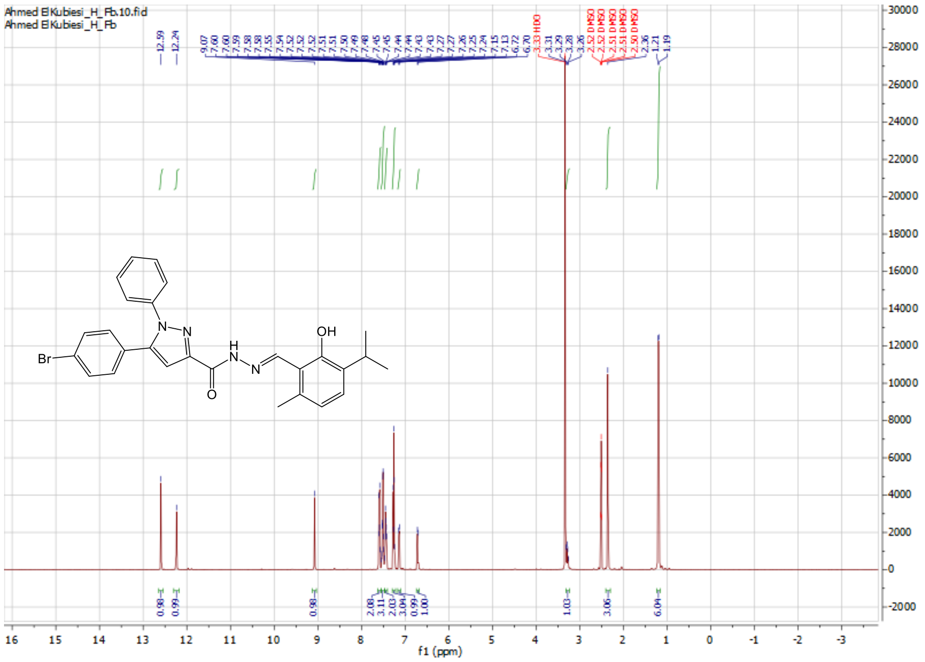
Ahmed El Kubiesi_H_Fb.10.fid
Ahmed El Kubiesi_H_Fb
f1 (ppm)

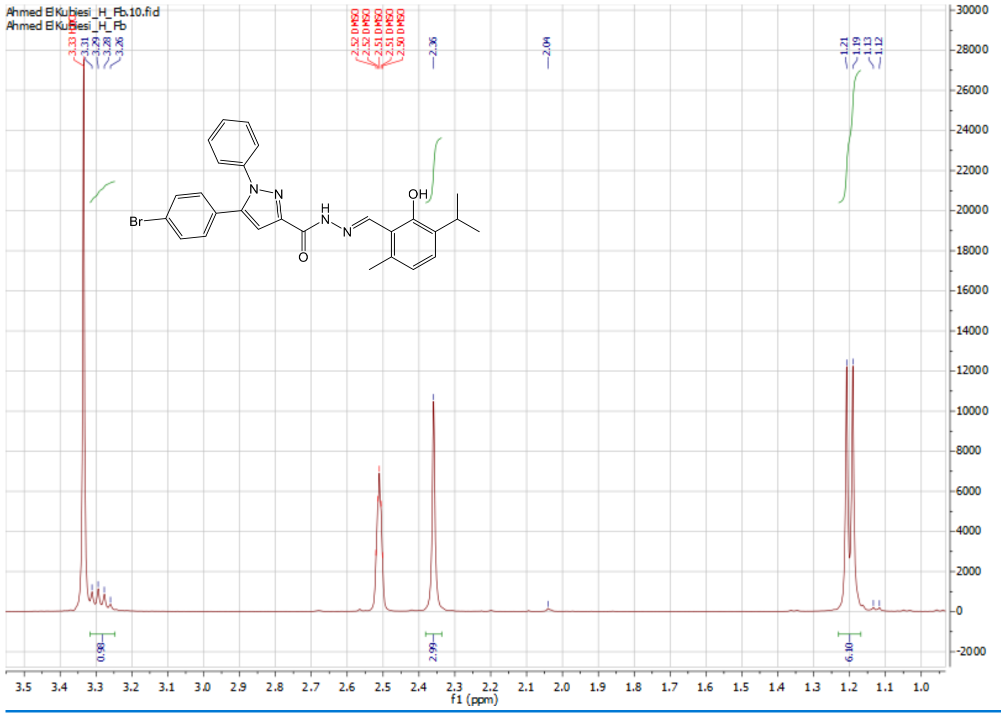
Ahmed El Kubiesi_H_Fb.10.fid
Ahmed El Kubiesi_H_Fb
f1 (ppm)

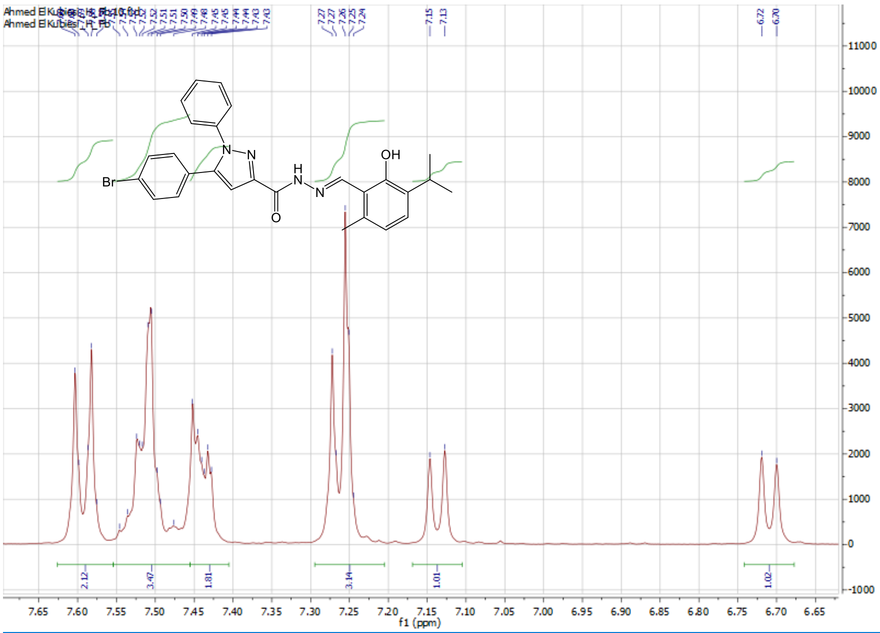

H-NMR of Compound 8e

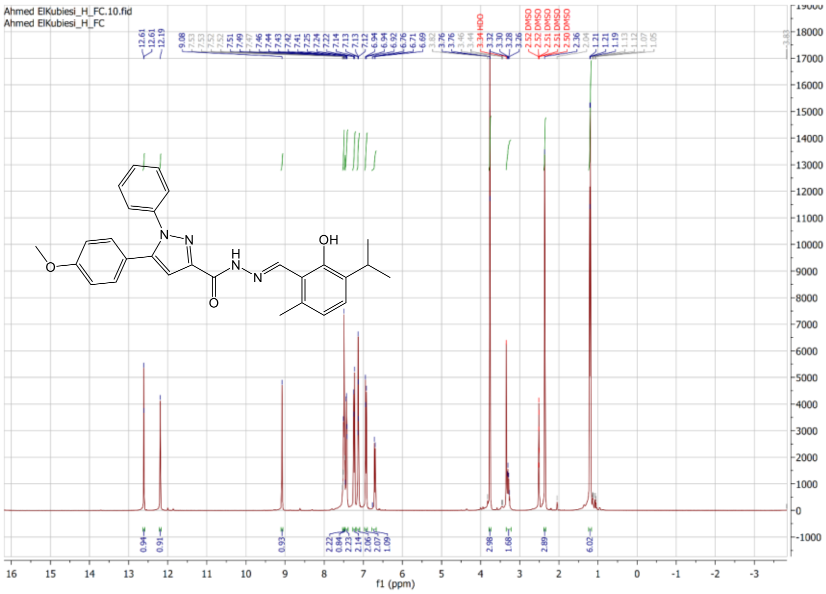

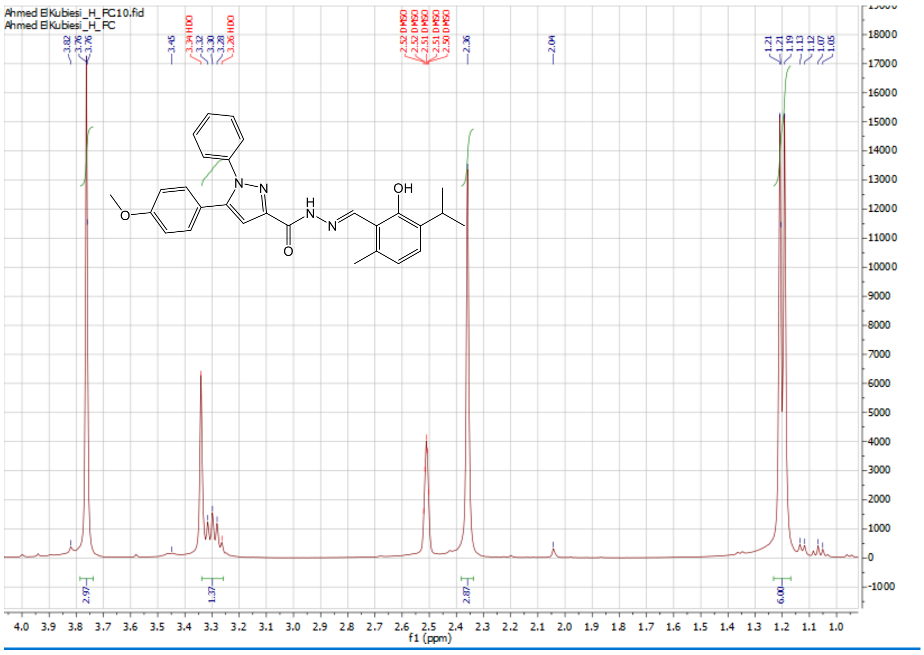

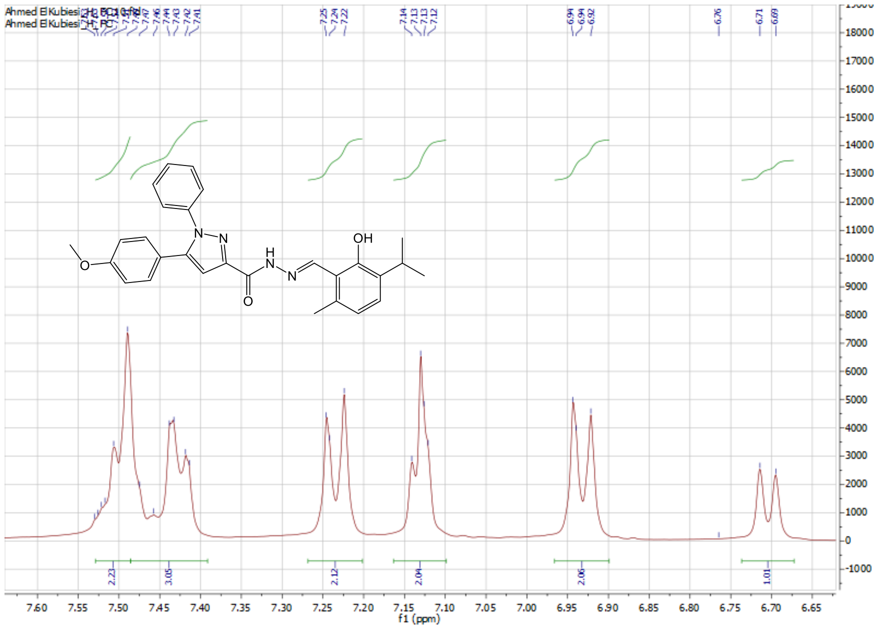

H-NMR of Compound 8f

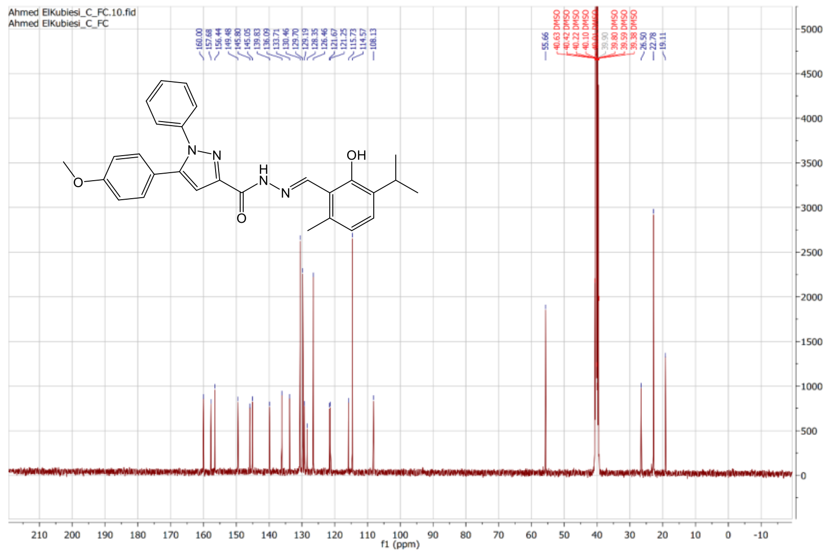

13C-NMR of Compound 8f

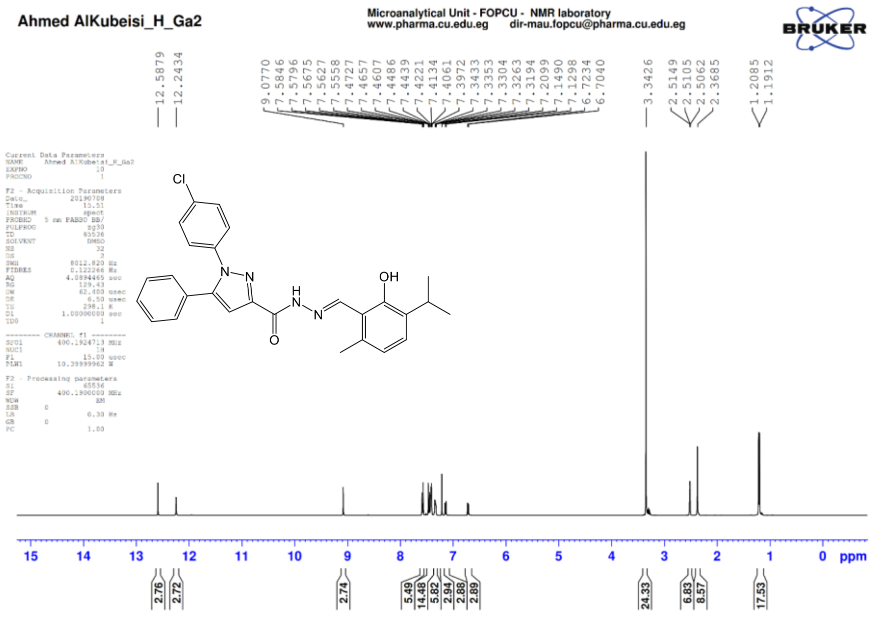

H-NMR of Compound 8g

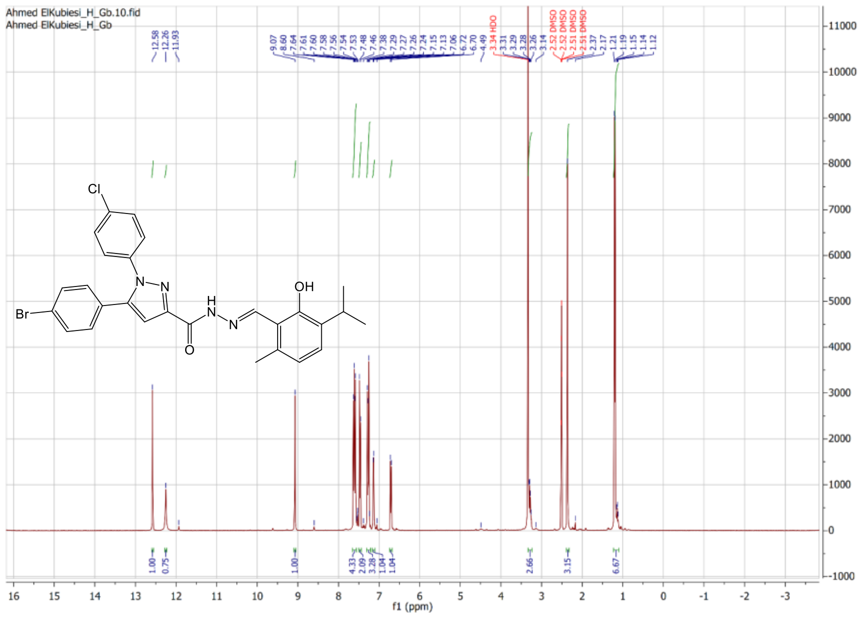

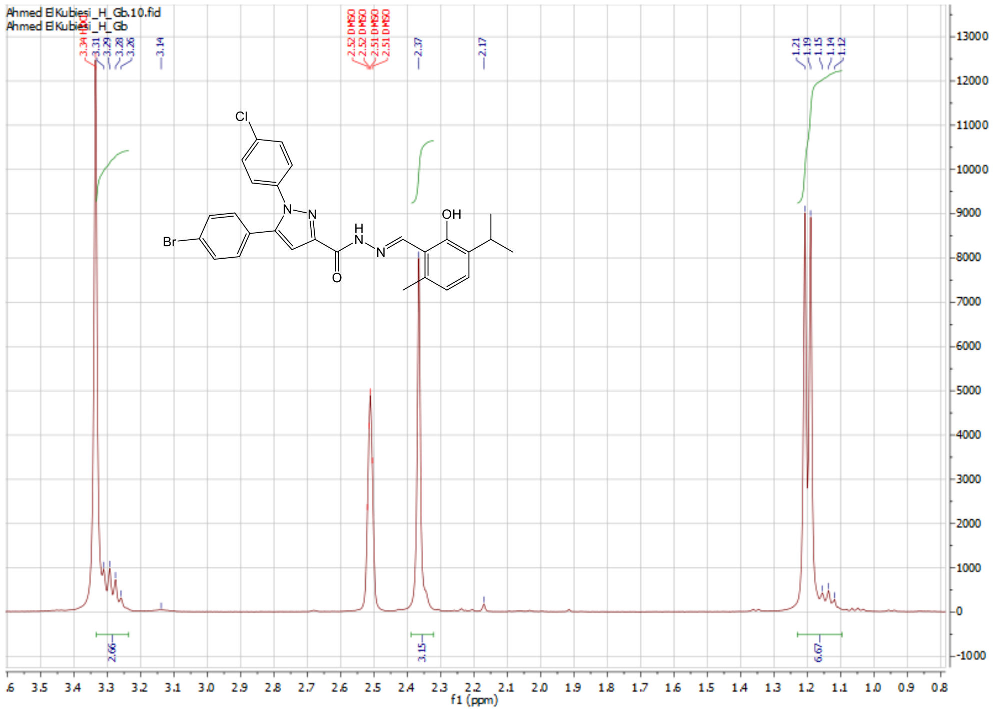

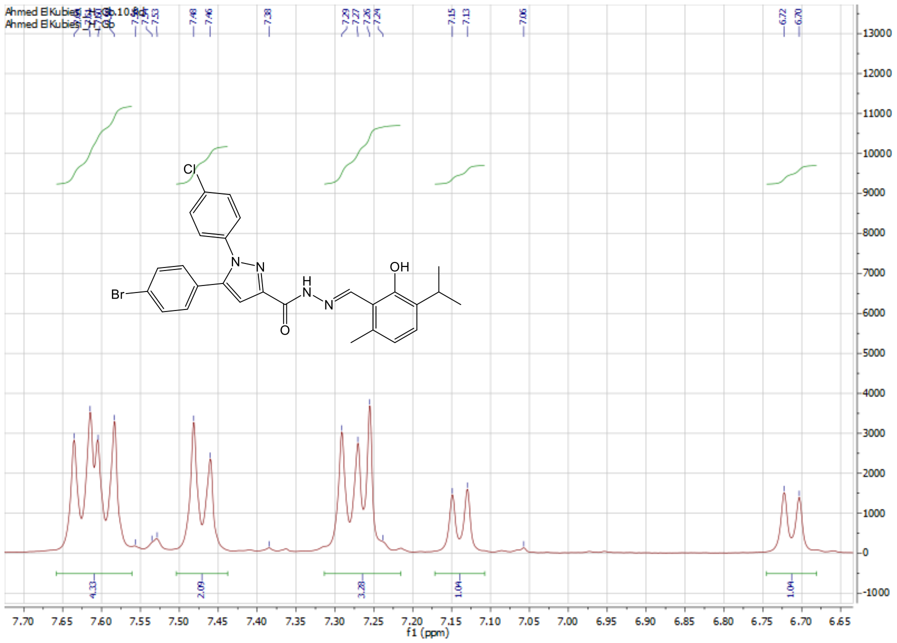

H-NMR of Compound 8h

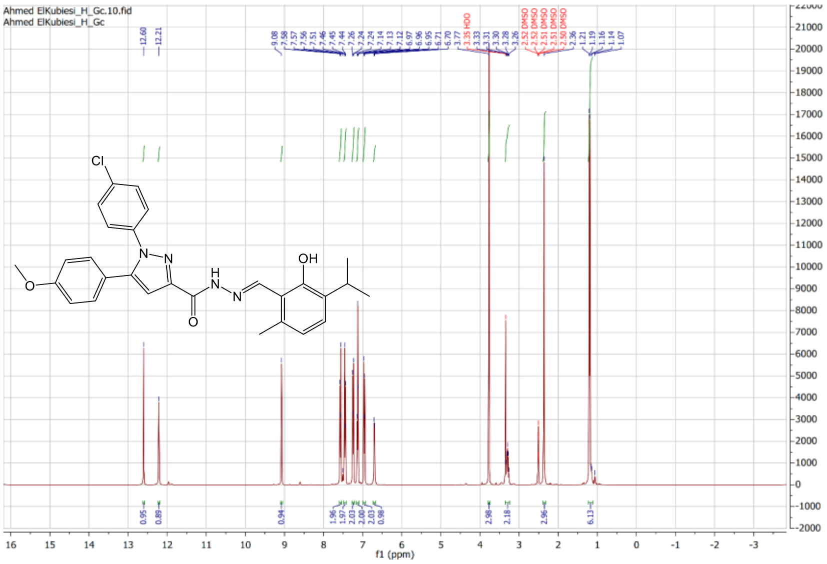

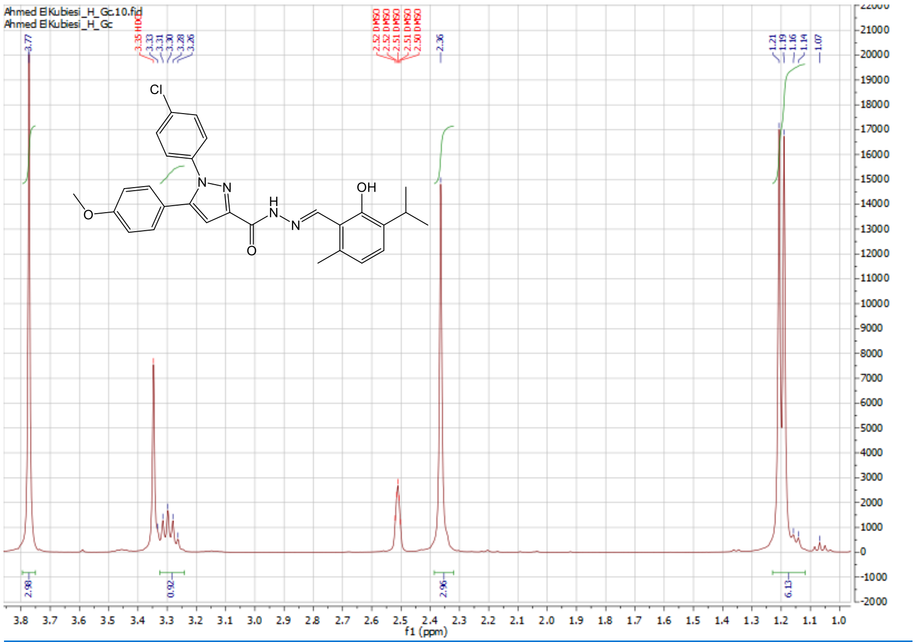

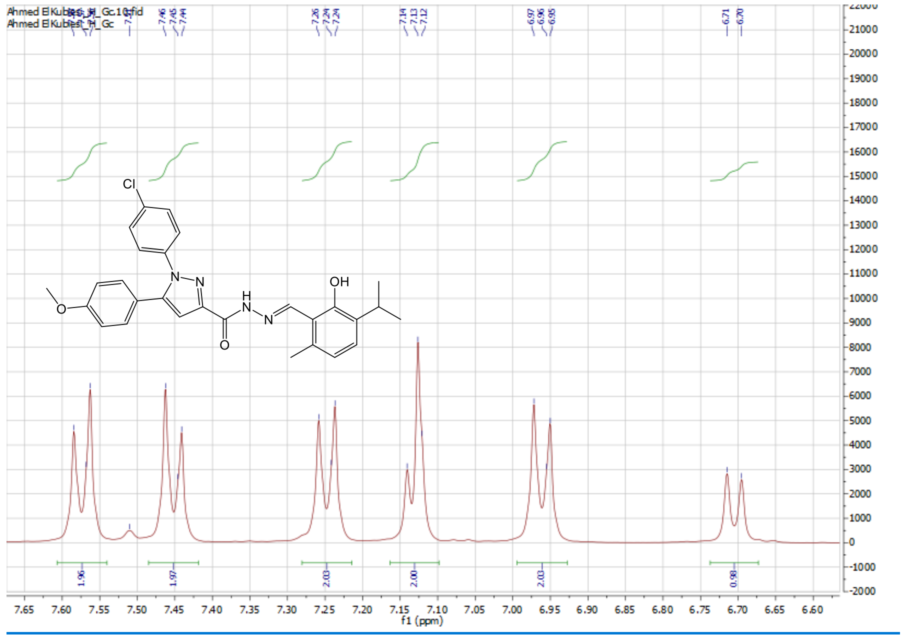

H-NMR of Compound 8i

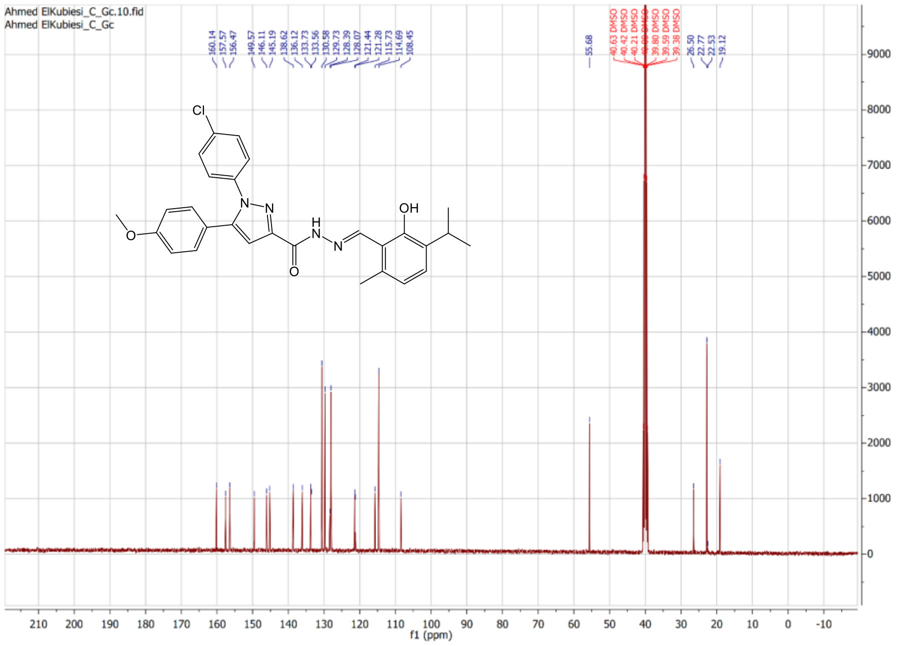

13C-NMR of Compound 8i

Biological Screening

Anti-inflammatory activity

***In vitro* COX-1 and COX-2 inhibitory assay**

Compounds **4a-c** and **8a-i** were screened for their ability to inhibit COX-1 and COX-2 enzymes in vitro. This was carried out using Cayman colorimetric COX (ovine) inhibitor screening assay kit (Catalog No. 560131) supplied by Cayman chemicals, Ann Arbor, MI, USA according to reported method [1]. The colorimetric COX inhibitor screening assay utilizes the peroxidase component of cyclooxygenase. The peroxidase activity was assayed calorimetrically by monitoring the appearance of oxidized *N*,*N*,*N'*,*N'*-tetramethyl-1,4-phenylenediamine (TMPD) at 590nm. The appearance of reagent was carried out following the instructions given with the assay kit (Catalog No. 560131). The half- maximal inhibitor concentrations (IC_{50}µM) were determined and the selectivity index (SI) values were calculated as IC_{50} (COX-1)/IC_{50} (COX-2).

Procedure:

1) **Background wells**- 160 µL of assay buffer and 10 µL of heme were added to background wells.
2) **The 100% Initial activity wells**- 150 µL of assay buffer, 10 µL of heme and 10 µL of enzyme (COX-1 or COX-2) were added.
3) Inhibitor well- 150 µL of assay buffer, 10 µL of heme and 10 µL of enzyme were added.
4) 10 µL of inhibitor (3 concentrations) in DMSO was added to the inhibitor wells to yield a final concentration of 25, 50 and 100 µM.

5) 10 µL of solvent (DMSO) was added to the 100% Initial activity wells and background wells.

6) The plate was shaken for a few seconds and incubated for 5 min at 25°C.

7) 20 µL of colorimetric substrate solution (TMPD) was added to all wells.

8) 20 µL of arachidonic acid was added to all wells to yield a final concentration of 100 µM.

9) The plate was shaken for a few seconds and incubated for 5 min at 25°C.

10) The absorbance was measured at 590 nm using a plate reader.

The calculations were carried out in the following manner:

1) The average absorbance of all samples was determined.

2) The absorbance of background wells was subtracted from absorbance of the 100% Initial activity and the inhibitor wells.

3) Percent inhibition = (100% Initial activity – Inhibitor wells/ 100% Initial activity)∗ 100

In vitro LOX inhibitory assay

Compounds **4a-c** and **8a-i** were screened for their ability to inhibit lipoxygenase enzymes. This was carried out using Abnova lipoxygenase inhibitor screening assay kit (Catalog No. 760700).[2] The Lipoxygenase Inhibitor Screening Assay Kit detects and measures the hydroperoxides produced in the lipoxygenation reaction using a purified LOX. The detection reaction is equally sensitive to hydroperoxides at various positions within the fatty acid and will work with fatty acids of any carbon length. It is thus a general detection method for 5-LOX and can be used to screen libraries of compounds for those which inhibit 5-LOX enzymes.

Procedure:

1) **Blank Wells**- add 100 µL of assay buffer to at least two wells.
2) **Positive Control Wells**- Lipoxygenase (15-LO standard) add 90 µL 15-LO and 10 µL of assay buffer to at least two wells.
3) 100% Initial Activity Wells- add 90 µL 15-LO and 10 µL of solvent (DMSO) (the same solvent to dissolve the inhibitor) to two wells. The 100% initial activity wells should result in approximately 10 nmol/min/mL of activity.
4) Inhibitor Wells- add 90 µL of sample and I10 µL of inhibitor to two wells.
5) Initiate the reaction by adding 10 µL of substrate (either arachidonic acid or linoleic acid) to all the wells. Place the 96-well plate on a shaker for at least five minutes.
6) Add 100 µL of chromogen to each well to stop enzyme catalysis and develop the reaction. Cover with a plate cover and place the 96-well plate on a shaker for at least five minutes.
7) Remove the cover and read the absorbance at 490-500 nm using a plate reader.

Inhibitors were dissolved in DMSO. The inhibitors were added to the assay in a final volume of 10 µL before initiating with substrate. Three concentrations were prepared (25, 50 and 100 µM) to determine the concentration produced 50% enzyme inhibition.

The calculations were carried out in the following manner:

1) The average absorbance of all samples was determined.
2) The absorbance of blank wells was subtracted from absorbance of the 100% Initial activity and the inhibitor wells.
3) Percent inhibition = (100% Initial activity – Inhibitor wells/ 100% Initial activity)∗ 100

In vivo anti-inflammatory activity

Approved by AlexU-IACUC. [3]

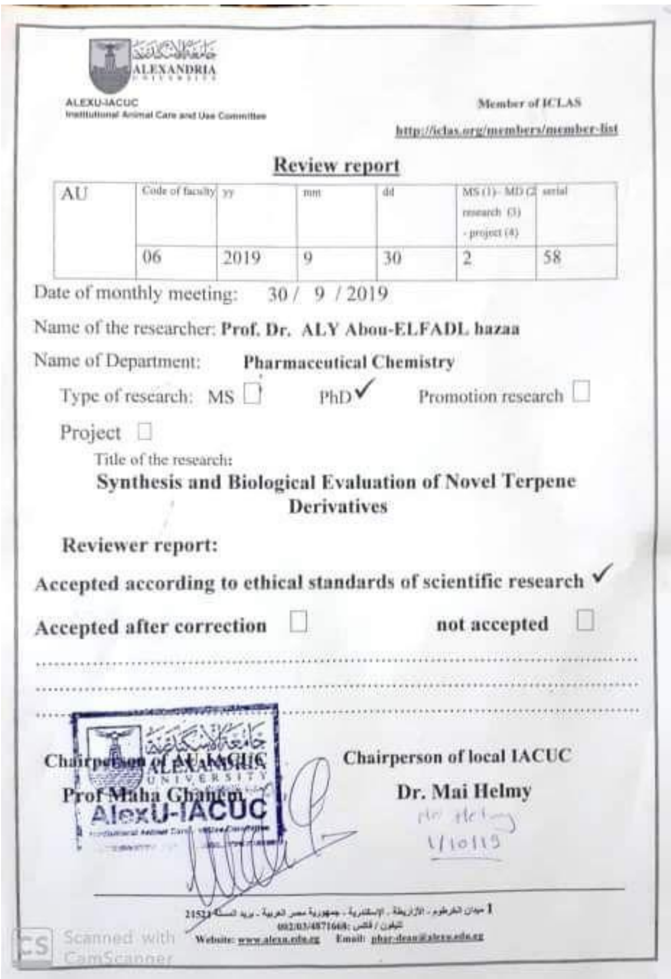

ALEXANDRIA UNIVERSITY

ALEXU-IACUC
Institutional Animal Care and Use Committee

Member of ICLAS
http://iclas.org/members/member-list

Review report

AU	Code of faculty	yy	mm	dd	MS (1)- MD (2) research (3) - project (4)	serial
	06	2019	9	30	2	58

Date of monthly meeting: 30 / 9 / 2019

Name of the researcher: **Prof. Dr. ALY Abou-ELFADL hazaa**

Name of Department: **Pharmaceutical Chemistry**

Type of research: MS ☐ PhD ✓ Promotion research ☐

Project ☐

Title of the research:
Synthesis and Biological Evaluation of Novel Terpene Derivatives

Reviewer report:

Accepted according to ethical standards of scientific research ✓

Accepted after correction ☐ **not accepted** ☐

Chairperson of AU IACUC
Prof Maha Ghanem

Chairperson of local IACUC
Dr. Mai Helmy

Dr. Helmy
1/10/19

1 ميدان الخرطوم - الأزاريطة - الإسكندرية - جمهورية مصر العربية - بريد [illegible] 21521
التليفون / فاكس: 002/03/4871668
Website: www.alexu.edu.eg Email: phar-dean@alexu.edu.eg

Scanned with CamScanner

Animals:

Animals Adult female albino rats weighing 150-250 g were used (Experimental Animal Centre in Alexandria University). All animals accessed to food and water *ad libitum* and were housed in 12 h dark/light cycle in a controlled condition at 23-25^{0}C. They were allowed to acclimatize for 1 week prior to experimentation. Procedures involving animals and their care were conducted in conformity with the Guide for the Care and Use of Laboratory Animals published by US National Institute of Health[4] (NIH publication No. 83-23, revised) and following the ethical guidelines of Alexandria University on laboratory animals. In all tests, adequate considerations were adopted to reduce pain or discomfort of animals (Approved by AlexU-IACUC) [3].

.

Compounds:

Celecoxib and Diclofenac sodium (European Egyptian Pharmaceutical industries, Alexandria, Egypt), formalin 5% made from formaldehyde 37% and saline (Merck, Germany) were used. The novel compounds were synthesized based on the previously described methods. Compounds that showed *in vitro* selectivity indices higher or nearly equivalent to reference drugs towards COX 2 enzyme, were further evaluated for their *in vivo* anti-inflammatory activity applying the formalin-induced paw edema screening protocol as an acute inflammation model. [5, 6]Celecoxib (5 mg/kg) and Diclofenac sodium (5 mg/kg) were used as reference drugs. Animals were divided into groups of six rats each treated with test compounds. Groups treated with Celecoxib and Diclofenac sodium served as references and rats which were given the vehicle (DMSO) served as control.

Formalin-induced paw edema test (acute inflammation model):

Procedure:

A solution of freshly prepared formalin 5% was used as a phlogistic agent. A mark was made on the lateral malleolus of the rats' paws delineating the injection sites. The initial volume of paw was measured by means of digital calibrated Vernier caliper. Then, the novel test compounds (5 mg/kg body weight), Celecoxib (5 mg/kg body weight), Diclofenac sodium (5 mg/kg body weight), or DMSO, as the control solution, were administered orally. After 45 minutes, 40 µl formalin were injected subcutaneously into the sub plantar tissue of the right hind paw of all groups under light ether anesthesia. An equal volume of saline was injected into the left hind paw and served as internal control for the degree of inflammation in the right hind paw. [5, 6] The volume of paw was measured in different treatment groups, 4 h following the formalin injection and the amount of increase in paw volume (edema volume) was calculated by subtracting the volumes before and 4 h after the injection of formalin. Edema was expressed as an increase in the volume of paw, and the percentage of edema inhibition (or percent protection against inflammation) for each rat and each group was calculated according to the following equation:

$$\%\ \text{Inhibition} = \frac{(Vt - Vo)\ \text{control} - (Vt - Vo)\ \text{test compound}}{(Vt - Vo)\ \text{control}} \times 100$$

Where Vt is the mean volume of edema at specific time interval (4 h) and Vo is the mean volume of edema at zero-time interval.

Relative potency of the tested compounds was expressed as % inhibition of edema for the tested compounds relative to % inhibition of edema for the reference drugs at 4 h from the induction of inflammation.

$$\%\ \text{Relative potency} = \frac{\%\ \text{inhibition of edema for the test compound after 4h} \times 100}{\%\ \text{inhibition of edema for the reference after 4h}}$$

(Approved by AlexU-IACUC) [3].

Gastric ulcerogenic activity:

Procedure:

Compounds were evaluated for acute gastric ulcerogenic effect in adult female Wistar rats. Rats (150–250 g) were divided into groups of six rats each and test compounds, references or DMSO as control were administered orally at a dose of 60 mg/kg body weight (three times the previously used dose). Six hours after the treatment they were sacrificed under deep ether anesthesia and their stomachs were removed and opened through greater curvature, washed under running water and fixed in saline solution. Gross examination was performed for any evidence of hyperemia, hemorrhage, definite hemorrhagic erosion or ulcer [6, 7] (Approved by AlexU-IACUC) [3].

Ulcerogenic activity:

The degree of ulcerogenicity was determined by viewing the gastric epithelial ulceration using a 5x magnifying lens and rated by ulcer score.

Ulcer score was used to grade the incidence and severity of the lesions such as:

1) Shedding of epithelium—10
2) Petechial and frank hemorrhages—20
3) One or more ulcers—30
4) More than two ulcers—40
5) Perforated ulcers—50

Molecular Modeling

The molecular modeling studies were performed using the Molecular Operating Environment (**MOE 2016.08**) software (Chemical Computing Group, Montreal, Canada). [8] and the crystal structures of the proteins were downloaded from the Protein Data Bank (PDB) website.

Steps for preparation of the compounds and enzymes for docking:
The ligand molecules were constructed using the builder module in MOE and collected in a database. The database was adapted by using the option "Protonate 3D" to add hydrogens, calculate partial charges and minimize energy (using Force Field MMFF94x). In addition, the downloaded proteins were prepared by deleting the repeated chains, water molecules and any surfactants, then hydrogens were added to the atoms of the receptor and the partial charges were calculated. The protocol in the MOE application was used to calculate the best score between the ligands and the enzymes' binding sites using triangle matcher as placement method and London dG as the scoring function. The output database contained the energy scores between the ligands' conformers and the enzyme binding sites in kcal/mol. The docking poses for each ligand were visually examined and the interactions with active site residues were analyzed. The highest scoring pose was selected to compute the ligand interactions using the Ligand Interactions module in MOE.
To confirm the validity of our docking results, the pose selection method was adopted to validate our docking protocol [9]. For all the used proteins, their co-crystallized ligand were drawn in MOE, prepared as the targeted compounds (hydrogens, partial charges and energy minimization), and then docked into the active site of the protein using our protocol. The Root Mean Square Deviation (RMSD) between the original and docked conformers was less than 2 Å for all the ligands. It was reported that values less than 2 Å were a sign of a successful and reliable docking protocol [9].

Formatted: Right: 0 cm, Space Before: 0 pt, After: 8 pt, Line spacing: single

Crystal structures of Cyclooxygenase-2 enzyme co-crystallized with Celecoxib (PDB: 3LN1) and 5-LOX in complex with Arachidonic acid (PDB: 3V99) were used for prediction of mode of binding of the active anti-inflammatory compounds.

REFERENCES

.1Cox inhibitor screening assay kit. No. 10006270.

.2Cayman C. Lipoxygenase inhibitor screening assay kit 760700. 201420.(

.3Hazzaa AA-E. Synthesis and biological evaluation of novel terpene derivatives. AlexU-IACUC (Member of ICLAS) 2019:AU-06-2019-9)58-2-30-

.4Council NR. Guide for the care and use of laboratory animals. National Academies Press.

.5Razmi A, Zarghi A, Arfaee S, Naderi N, Faizi M. Evaluation of anti-nociceptive and anti-inflammatory activities of novel chalcone derivatives. Iranian journal of pharmaceutical research: IJPR 2013:12(Suppl): 153.

.6Lakshmi V, Mishra V, Palit G. A new gastroprotective effect of limonoid compounds xyloccensins x and y from xylocarpus molluccensis in rats. Natural products and bioprospecting 2014:4(5): 277.283-

.7Srivastava S, Nath C, Gupta M, Vrat S, Sinha J, Dhawan K, Gupta G. Protection against gastric ulcer by verapamil. Pharmacological research 1991:23(1): 81-86.

.8Molecular operating environment (moe) 2016.08, chemical computing group inc. 1010 sherbrooke st. West, suite #910, montreal, qc, canada, h3a2r7. Www.Chemcomp.Com.

.9Hevener KE, Zhao W, Ball DM, Babaoglu K, Qi J, White SW, Lee RE. Validation of molecular docking programs for virtual screening against dihydropteroate synthase. Journal of Chemical Information and Modeling 2009:49(2): 444-460.

Formatted: Left
